# Amino Acid Metabolic Vulnerabilities in Acute and Chronic Myeloid Leukemias

**DOI:** 10.3389/fonc.2021.694526

**Published:** 2021-07-01

**Authors:** Aboli Bhingarkar, Hima V. Vangapandu, Sanjay Rathod, Keito Hoshitsuki, Christian A. Fernandez

**Affiliations:** ^1^ Center for Pharmacogenetics and Department of Pharmaceutical Sciences, University of Pittsburgh School of Pharmacy, Pittsburgh, PA, United States; ^2^ Division of General Internal Medicine, University of Pittsburgh School of Medicine, Pittsburgh, PA, United States

**Keywords:** non-essential amino acid, GCN2, general control non-derepressible 2, mTORC1, myeloid leukemias

## Abstract

Amino acid (AA) metabolism plays an important role in many cellular processes including energy production, immune function, and purine and pyrimidine synthesis. Cancer cells therefore require increased AA uptake and undergo metabolic reprogramming to satisfy the energy demand associated with their rapid proliferation. Like many other cancers, myeloid leukemias are vulnerable to specific therapeutic strategies targeting metabolic dependencies. Herein, our review provides a comprehensive overview and TCGA data analysis of biosynthetic enzymes required for non-essential AA synthesis and their dysregulation in myeloid leukemias. Furthermore, we discuss the role of the general control nonderepressible 2 (GCN2) and-mammalian target of rapamycin (mTOR) pathways of AA sensing on metabolic vulnerability and drug resistance.

## Introduction

Myeloid leukemias are a group of disorders characterized by the presence of increased numbers of immature myeloid cells in the marrow and peripheral blood. These diseases are most common in adults with survival rates and first-line therapy options varying by age. In this review, we focus on the differences of non-essential amino acid (NEAA) metabolism in myeloid leukemias with an emphasis on the enzymes required for amino acid biosynthesis. While normal cells can synthesize NEAAs, cancers, in order to maintain their rapid growth and proliferation, can alter the expression of genes involved in amino acid biosynthesis and therefore lose the ability to synthesize specific NEAAs. This auxotrophy, or the inability of cancer cells to synthesize certain NEAA amino acids required for growth, leads to a dependency on exogenous sources of NEAAs, which can be pharmacologically targeted.

To comprehensively examine potential metabolic targets in myeloid leukemias within this review, we first introduce the disease and currently available pharmacotherapies to demonstrate the need for novel agents and to identify patients most likely to benefit from pharmacological agents targeting NEAA dependencies. We thereafter discuss the biosynthesis of NEAA, explore gene expression data analysis of potentially dysregulated pathways, and consider the supporting evidence for novel vulnerabilities in myeloid leukemias. Lastly, we detail mechanisms of cancer cell adaptation to nutrient stress and considerations for the optimal design of strategies targeting NEAA metabolic vulnerabilities.

## Myeloid Leukemias and Need for Novel Treatment Options

### Novel Drug Therapies Are Needed to Improve Outcomes in Myeloid Leukemia Patients

Myeloid leukemias are classified as either chronic or acute based on their proliferation rate. Acute and chronic myeloid leukemias make up 14 and 33% of all estimated new 2020 leukemia cases in the United States, respectively (1).

Acute myeloid leukemia (AML) is the most common acute leukemia in adults. As of 2016, the 5-year overall survival (OS) of AML is approximately 24% (2), but the highest rate of AML deaths in the United States is among older patients (~90% for ages ≥ 65)_1_, underscoring the need for better therapy options. Due to the lower performance status of this demographic and inability to tolerate aggressive chemotherapy, these older patients have more limited curative pharmacotherapy options available.

Younger AML patients with good performance status can receive traditional intensive remission-induction chemotherapy with cytarabine, an anthracycline, and a FLT3 inhibitor depending on FLT3 mutation status. Alternatively, pharmacotherapy options for patients not eligible for intensive induction therapy include targeted approaches such as the hypomethylation agents decitabine or azacitidine and the BCL2 inhibitor venetoclax (3). Current approaches for post-induction therapy for low-risk patients generally include additional therapy using similar induction agents (such as high-dose cytarabine), and higher risk patients proceed to allogenic hematopoietic cell transplantation (HCT).

The main characteristic of chronic myeloid leukemia (CML) is the translocation between chromosomes 22 and 9 (i.e. Philadelphia chromosome) that creates a *BCR-ABL* fusion oncogene with constitutively activated tyrosine kinase activity (4, 5). The cornerstone of CML treatment is the *BCR-ABL* tyrosine kinase inhibitor (6). However, there remains a need to develop novel therapies for CML patients with unfavorable prognosis, including those that are negative for the Philadelphia chromosome (7) and the 20–30% of CML patients who fail to achieve treatment milestones or develop TKI resistance. Altogether, current responses to standard AML and CML treatment regimens indicate that while many patients attain responses, there remains a need to develop therapies for those patients with poor prognosis.

### Targeting AAs Dependencies in Myeloid Leukemias

Novel agents for the treatment of myeloid leukemias with unfavorable prognosis will most likely rely on certain cancer cell vulnerabilities to specifically inhibit their growth and limit treatment toxicity. Differences between the metabolic status of cancerous and normal cells are one of the primary hallmarks of cancer (8), and changes in AA metabolism, glucose utilization, lipid consumption, and ATP generation are adaptations cancer cells can acquire for meeting their increased energy demands (9–13). These metabolic changes can lead to an “addiction” of particular fuel sources and identification of these vulnerabilities has led to the development of several agents directed toward specific molecular targets (14).

AAs are the building blocks of protein and are necessary for the sustenance of both normal and leukemic cells. There are 20 AAs classified as essential, non-essential, and conditionally essential (15). Essential AAs must be obtained exogenously from diet, whereas the body possesses the metabolic pathways to catalyze the synthesis of NEAAs. Conditionally essential AAs, like arginine and glutamine, are those that can become essential during certain physiological conditions that limit their synthesis (16). AAs can serve as precursors to many biological compounds, including those involved nucleotide synthesis (17), redox balance (18), lipogenesis (19, 20), and molecules that can fuel the TCA cycle (21). Furthermore, AAs can modulate the tumor microenvironment (22), global chromatin structures (23, 24), and epigenetic factors (25–28).

Herein, our discussion concerning myeloid leukemias and amino acid metabolism will focus on identifying potential vulnerabilities in AA biosynthesis that could be exploited for therapeutic purposes. Below, we provide a thorough discussion on NEAA biosynthesis for subsequently identifying dysregulations in potential targets that can lead to a dependency on extracellular NEAA sources for survival. Upon identification of an AA dependency, there are several targets within AA metabolic pathways that can be selectively targeted to exploit the vulnerability, including AA transporters, synthases, and transaminases that regulate biosynthesis (13, 29–31). In addition, enzymatic AA depletion therapy is an effective strategy for preferentially targeting cancer cells dependent on a specific AA for maintaining cell proliferation demands (13).

## Biosynthesis of NEAA

### Glutamate Plays a Central Role in the Biosynthesis of Several NEAA

**Figure 1:**
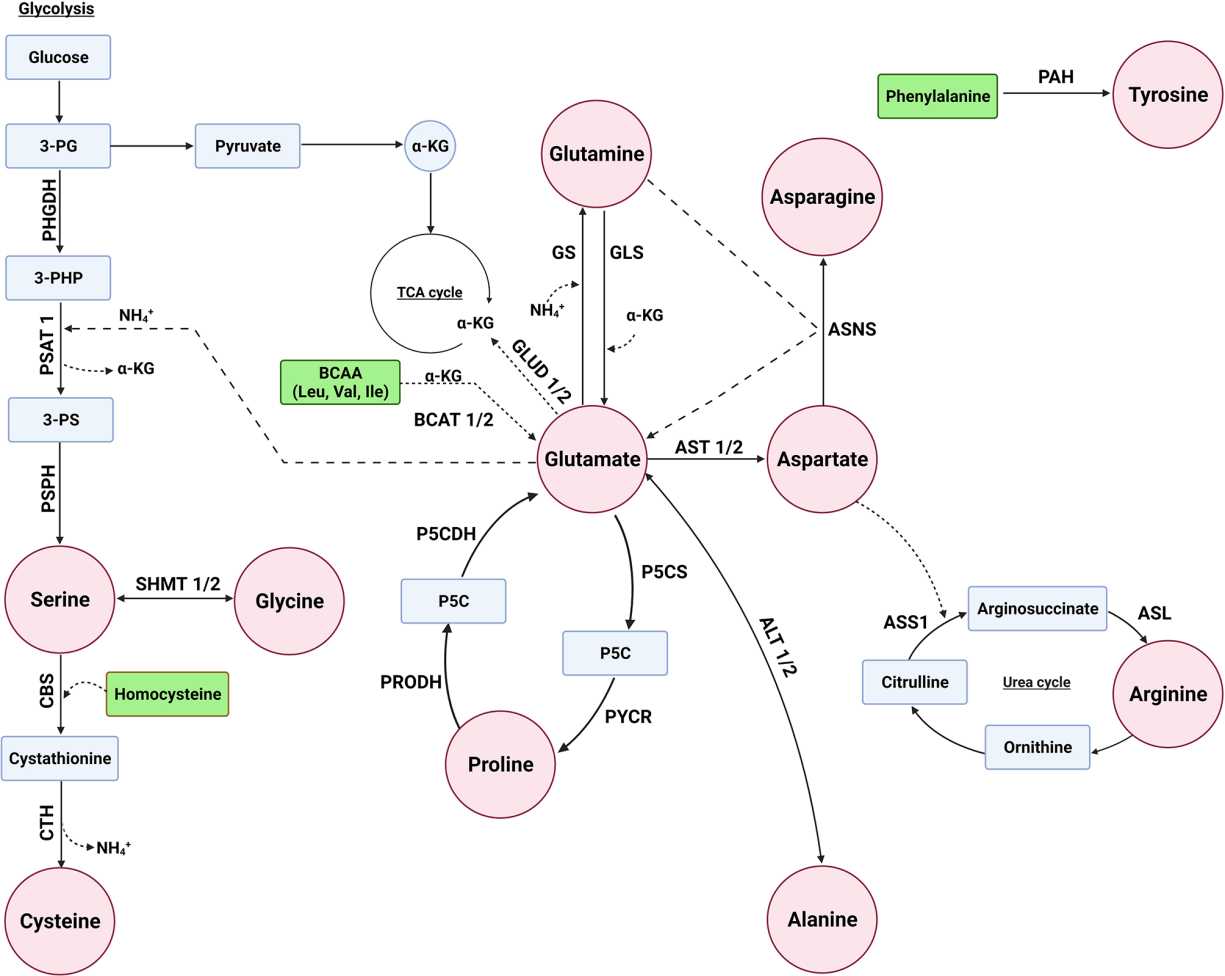
Several enzymes are involved in the biosynthesis of non-essential amino acids. Glutamate can serve as a precursor for the biosynthesis of alanine, aspartate, glutamine, proline, and serine. The enzymes glutaminase (GLS/GLS2), proline dehydrogenase 1 (PRODH), P5C dehydrogenase (P5CDH or ALDH4A1), and branched chain amino acid transaminase (BCAT1/2) are involved in glutamate biosynthesis, whereas glutamate dehydrogenas (GLUD1/2) is involved in its metabolism. The enzymes alanine aminotransaminase (ALT1/2 or GPT/2), aspartate aminotransferase (AST1/2 or GOT1/2), and glutamine synthetase (GS or GLUL) convert glutamate to alanine, aspartate, and glutamine, respectively. Pyrroline-5-carboxylate synthase (P5CS/ALDH18A1) and pyrroline-5-carboxylate reductase 1 (PYCR1) are used for proline biosynthesis from glutamate, whereas serine biosynthesis involves the transamination of 3-phosphohydroxypyruvate to 3-phosphoserine (3-PS) by phosphoserine aminotransferase (PSAT1) with glutamate as the amino donor and phosphoserine phosphatase (PSPH) to convert 3-PS to serine. Asparatate is a precursor of asparagine and arginine. The enzyme asparagine synthetase (ASNS), argininosuccinate synthase 1 (ASS1), and argininosuccinate lyase (ASL) are required for converting aspartate to asparagine and arginine, respectively. Serine is a precursor of glycine and cysteine, where serine is converted to glycine by serine hydroxymethyltransferase (SHMT1/2). Cysteine is synthesized through a process called the reverse transsulfuration pathway involving cystathionine β-synthase (CBS) and cystathionine γ-lyase (CTH). Tyrosine biosynthesis not linked to glutamate, but rather the enzyme phenylalanine hydroxylases (PAH) converts phenylalanine to tyrosine.

The concentration of glutamate in the body is tightly regulated to balance its important role in various biological processes (32). The biosynthesis of several NEAAs is interlinked with each other with glutamate playing a central role in the synthesis of several NEAAs as a precursor of alanine, proline, glutamine, aspartate, and serine (**Figure 1**). There are several biochemical pathways leading to the synthesis of glutamate, with a majority of this NEAA derived from the hydrolysis of glutamine by the amidohydrolase enzyme, glutaminase (GLS). Glutamate can also be derived from branch-chain amino acids (BCAAs) and proline (**Figure 1**). BCAA aminotransferase (BCAT) catalyzes the conversion of BCAAs and α-ketoglutarate into glutamate and branched chain α-keto acids, whereas oxidation of proline occurs *via* two enzymes: proline dehydrogenase (PRODH) and P5C dehydrogenase (P5CDH). In addition, glutamate is metabolized by glutamate dehydrogenase (GLUD1/2) back to α-ketoglutarate (33).

### Alanine, Aspartate, Glutamine, Proline, and Serine Can Be Synthesized From Glutamate

As previously mentioned, glutamate serves as a substrate for several enzymes involved in the synthesis of several NEAAs. For alanine, the enzyme alanine aminotransferase (ALT/GPT) catalyzes the transamination of pyruvate, with glutamate as the amino donor, to synthesize alanine (34). Similar to ALT, aspartate aminotransferase (AST/GOT) transfers an amine group from glutamate to oxaloacetate of the TCA cycle producing aspartate and α‐ketoglutarate (35).

Glutamine is the most abundant amino acid in blood and is indispensable for the survival and growth of cancers. While humans acquire glutamine mostly through diet, glutamine synthetase (GS or GLUL, **Figure 1**) can synthesize glutamine through the condensation of glutamate and ammonia (36). For proline biosynthesis (**Figure 1**), pyrroline-5-carboxylate synthase (P5CS) produces P5C from glutamate, which is converted to proline by pyrroline-5-carboxylate reductase (PYCR) (37).

Glutamate is also used for the biosynthesis of serine (**Figure 1**), where the glycolysis intermediate 3-phosphoglycerate (3-PG) is oxidized by phosphoglycerate dehydrogenase (PHGDH) to generate 3-phosphohydroxypyruvate (3-PHP). Phosphoserine aminotransferase (PSAT) catalyzes the transamination of 3-phosphohydroxypyruvate to 3-phosphoserine (3-PS) with glutamate as the amino donor, and phosphoserine phosphatase (PSPH) hydrolyzes 3-phosphoserine to serine (38).

### Aspartate Is a Precursor for the Biosynthesis of Asparagine and Arginine

Similar to glutamate, other NEAAs, such as aspartate, serve as precursors for the biosynthesis of other AAs. Asparagine is synthesized from aspartate and glutamine *via* asparagine synthetase (ASNS, **Figure 1**), which transfers an amine group of glutamine to aspartate (39). Similarly, aspartate can be used for the synthesis of arginine through a multi-enzyme pathway in which the enzyme argininosuccinate synthase 1 (ASS1) initiates the process by catalyzing the formation of argininosuccinate from citrulline, which is an intermediate of urea synthesis, and aspartate (**Figure 1**). Subsequent cleavage of argininosuccinate by argininosuccinate lyase (ASL) produces arginine and fumarate (40).

### Serine Is a Precursor of Both Cysteine and Glycine

Serine can serve as a precursor for several AAs, including cysteine and glycine (**Figure 1**). While cancers can dysregulate cysteine biosynthesis (41–43), typically cysteine is derived from extracellular sources (44–46). The metabolic pathway that leads to the generation of several sulfur metabolites, including cysteine, is called the transsulfuration pathway, where mammalian cells can only obtain cysteine through the reverse transsulfuration pathway (47–49). This pathway for generating cysteine in mammals involves the condensation of homocysteine (derived from methionine) with serine to generate cystathionine through the enzyme cystathionine β-synthase (CBS). Cystathionine γ-lyase (CTH) then converts cystathionine to cysteine (50).

Serine can also be converted to glycine by serine hydroxymethyltransferase (SHMT), which transfers the β-carbon of serine to tetrahydrofolate to produce glycine and 5,10-methylene tetrahydrofolate (51). In humans, there are two SHMT genes: cytoplasmic SHMT1 and mitochondrial SHMT2 (52).

### Tyrosine Is Synthesized by Phenylalanine Hydroxylases (PAH) From Phenylalanine

While the biosynthesis of other NEAA is linked to each other, mammals synthesize tyrosine from the essential amino acid phenylalanine. The enzyme phenylalanine hydroxylases (PAH) hydroxylates the aromatic side-chain of phenylalanine to generate tyrosine (53).

## Dysregulation of Enzymes Involved in NEAA Biosynthesis in Myeloid Malignancies

### Gene Expression Analysis of TCGA AML Samples

#### The Expression of Enzymes Involved in the Biosynthesis of Glutamate Is Dysregulated in AML

A gene expression analysis of enzymes involved in NEAA biosynthesis was performed using the Gene Expression Profiling Interactive Analysis (GEPIA) web server (54, 55). GEPIA uses RNA-Seq datasets based on the UCSC Xena project_2_ that has a standard processing pipeline allowing users to compare gene and transcript expression from The Cancer Genome Atlas (TCGA) tumor samples to corresponding Genotype-Tissue Expression (GTEx) normal samples. GEPIA includes over 8,000 normal samples from TCGA and the GTEx projects, albeit from unrelated donors (56, 57). For AML, GEPIA was used to screen for dysregulated enzymes by comparing transcript expression between AML (n = 173) and corresponding normal GTEx samples (n = 70).

**Figure 2:**
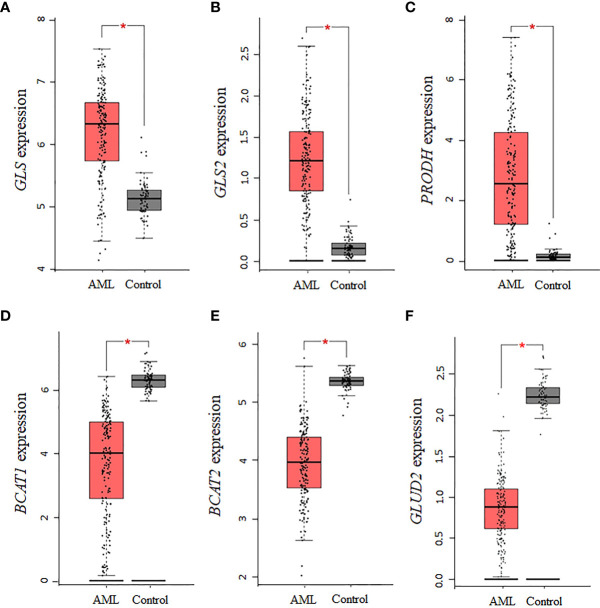
Several enzymes involved in the biosynthesis of glutamate are dysregulated in AML. The expression levels of genes involved in the biosynthesis and metabolism of glutamate were assessed in AML TCGA samples using GEPIA. The data indicate that the expression of **(A)** *GLS*, **(B)** *GLS2*, and **(C)** *PRODH* is upregulated, whereas the expression of **(D)** *BCAT1*, **(E)** *BCAT2*, and **(F)** *GLUD2* is downregulated. Log2 (TPM + 1) was used for log-scale and P values ≤ 0.01 are denoted with an asterisk (*) and indicate significance (n_AML_ = 173; n_control_ = 70).

Using GEPIA and TCGA AML samples, a gene expression analysis of enzymes involved in the synthesis of glutamate was performed. Consistent with the central role glutamate plays in the biosynthesis of several NEAA, we found that the expression of the glutaminase isoforms *GLS* and *GLS2* in AML is upregulated relative to normal controls (P <0.01, **Figures 2A, B**). Similar to *GLS*, *PRODH* was significantly upregulated in AML samples (P <0.01, **Figure 2C** **)**, whereas expression levels of *P5CDH* (i.e., *ALDH4A1*, data not shown) showed a similar but non-statistically significant trend. Interestingly, the two isoforms of *BCAT* (cytosolic *BCAT1* and mitochondrial *BCAT2*) were downregulated in AML TCGA samples (P <0.01, **Figures 2D, E** **)**, indicating a potential strong dependence on glutamine and proline for sources of glutamate. For glutamate dehydrogenase, which is involved in glutamate metabolism, no association was identified for *GLUD1*, whereas the *GLUD2* genes was significantly downregulated **(**P <0.01, **Figure 2F** **)**, consistent with a glutamate dependency in AML.

#### The Expression of Enzymes Involved in the Biosynthesis of NEAA Requiring Glutamate as a Precursor Is Decreased in AML

**Figure 3:**
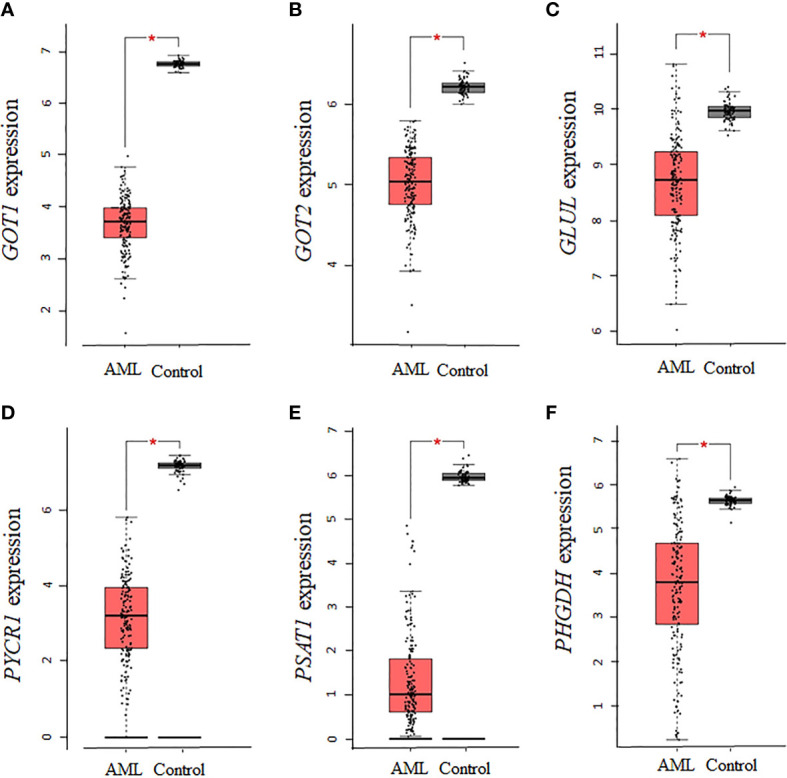
The expression of genes involved in the biosynthesis of alanine, aspartate, glutamine, proline, and serine is decreased in AML. The expression of genes involved in the biosynthesis of alanine, aspartate, glutamine, proline, and serine was analyzed using TCGA AML samples. **(A)**, *GOT1*, **(B)** *GOT2*, **(C)** *GLUL*, **(D)** *PYCR1*, **(E)** *PSAT1*, and **(F)** *PHGDH* gene expression was significantly downregulated in AML. Log2 (TPM + 1) was used for log-scale and P values ≤ 0.01 are denoted with an asterisk (*) and indicate significance (n_AML_ = 173; n_control_ = 70).

Given that several enzymes of glutamate biosynthesis were dysregulated in AML, it was possible that the corresponding effect on glutamate levels in AML had an influence on other enzymes involved in the biosynthesis of NEAAs requiring glutamate as a precursor. An analysis of the 10 enzymes involved in the biosynthesis of alanine, aspartate, glutamine, proline, and serine identified significant downregulations in *AST* (*GOT1* and *GOT2*), *GLUL*, *PYCR1*, *PSAT1*, and *PHGDH* (P <0.01, **Figures 3A–F**), but no statistical dysregulations in *ALT* (*GPT* and *GPT2*), *P5CS* (*ALDH18A1*), or *PSPH* (data not shown). Overall, there was a downregulation in 6 of 10 enzymes involved in the biosynthesis of these NEAAs, with alanine being the only NEAA with no association. Therefore, while there seems to be a dependence on glutamine and proline for sources of glutamate, many of the pathways involved in the biosynthesis of NEAA requiring glutamate as precursor were repressed.

#### The Expression of Enzymes Involved in the Biosynthesis of NEAA Requiring Aspartate or Serine as Precursors Is Decreased in AML

**Figure 4:**
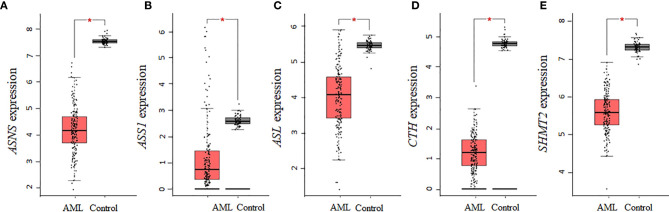
The gene expression of enzymes involved in the biosynthesis of asparagine, arginine, cysteine, and glycine is decreased in AML. AML TCGA analysis of genes involved in the biosynthesis of asparagine and arginine identified a statistical downregulation in **(A)** *ASNS*, **(B)** *ASS1*, and **(C)** *ASL*. Analysis of genes involved in cysteine and glycine biosynthesis identified a statistical downregulation in **(D)** *CTH* and **(E)** *SHMT2*. Log2 (TPM + 1) was used for log-scale and P values ≤ 0.01 are denoted with an asterisk (*) and indicate significance (n_AML_ = 173; n_control_ = 70).

Aspartate is a precursor for the biosynthesis of asparagine and arginine, whereas serine is a precursor of cysteine and glycine. Of the seven collective enzymes involved in the biosynthesis of asparagine, arginine, cysteine, and glycine, we found that *ASNS*, *ASS1*, *ASL*, *CTH*, and *SHMT2* were all statistically downregulated in AML samples (**Figures 4A–E**), whereas *CBS* and *SHMT1* showed non-statistically significant downregulations relative to controls (data not shown). Altogether, our analysis using the AML samples available through TCGA shows that there is a consistent decrease in the expression of enzymes involved in the biosynthesis of NEAAs that are interconnected through glutamate. In contrast, for tyrosine, whose biosynthesis is not linked to glutamate, there was no statistical difference in the expression of *PAH* between AML and control samples.

In summary, using GEPIA and TCGA AML samples we identified statistically significant dysregulations in enzymes involved in the biosynthesis of glutamate, aspartate, glutamine, proline, serine, asparagine, arginine, cysteine, and glycine. Therefore, future efforts should focus on validating these associations across various AML subtypes to best identify specific patient groups with potential NEAA metabolism vulnerabilities, which was not possible using GEPIA.

### Gene Expression Analysis of NEAA Biosynthesis in CML

#### The Expression of Enzymes Involved in Regulating Glutamate, Aspartate, Alanine, Asparagine, and Cysteine Levels Is Upregulated in CML

**Figure 5:**
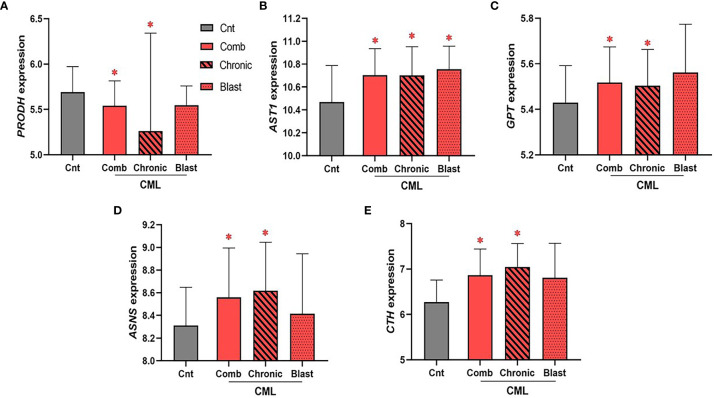
The expression of enzymes involved in glutamate, aspartate, alanine, asparagine, and cysteine biosynthesis is dysregulated in CML. The expression of 26 genes encoding enzymes involved in the biosynthesis of NEAA was analyzed in combined (“comb”) and phase disaggregated (“chronic” and “blast” phase) CML samples. **(A)** We identified a significant downregulation in *PRODH* expression in combined (P_Comb_ value = 4.6 × 10^−2^) and chronic (P_Chronic_ value = 9.4 × 10^−3^) CML samples. In contrast, **(B)** *AST1* (P_Comb_ value = 0.02; P_Chronic_ value = 0.03), **(C)** *GPT/ALT1* (P_Comb_ value = 0.03; P_Chronic_ value = 0.04), **(D)** *ASNS* (P_Comb_ value = 0.04; P_Chronic_ value = 0.02), and **(E)** *CTH* (P_Comb_ value = 3 × 10^−4^; P_Chronic_ value < 1 × 10^-4^) were all upregulated in the combined and chronic CML sample analysis. Only *AST1* expression was significantly dysregulated in blast CML samples (P_Blast_ value = 4.8 × 10^−2^). Log2 was used for log-scale and differential expression was determined using Mann–Whitney testing (n_Cnt_ = 15, n_Comb_ = 52, n_Chronic_ = 24, and n_Blast_ = 10). P values < 0.05 are denoted with an asterisk (*).

CML samples were not available through the TCGA database; rather, we used the largest publicly available data set disaggregated by CML phase with gene expression for all 26 genes involved in the biosynthesis of NEAA (Gene Expression Omnibus, GEO accession no. GSE47927)_3_. Unlike AML, we found few statistical associations for genes encoding enzymes involved in NEAA biosynthesis (**Figure 5**). Of the 26 genes encoding enzymes involved in the biosynthesis of NEAAs used for our AML analysis, only five statistically significant dysregulated genes were identified in the combined CML cohort. We identified a significant downregulation in *PRODH* expression in combined and chronic phase CML samples (**Figure 5A**, P_Comb_ = 4.6 × 10^−2^, P_Chronic_ = 9.4 × 10^−3^), and significant upregulations in *AST1* (**Figure 5B**, P_Comb_ = 0.02, P_Chronic_ = 0.03), *GPT/ALT1* (**Figure 5C**, P_Comb_ = 0.03; P_Chronic_ = 0.04), *ASNS* (**Figure 5D**, P_Comb_ = 0.04; P_Chronic_ = 0.02), and *CTH* (**Figure 5E**, P_Comb_ = 3 × 10^−4^; P_Chronic_ <1 × 10^−4^) in the combined and chronic phase CML sample analysis. Of the genes that were identified as significant in the combined CML analysis, only *AST1* upregulation was significant in blast phase CML samples (**Figure 5B**, P_Blast_ = 4.8 × 10^−2^). While no consistent similarities between AML and CML were identified, dysregulation of genes involved in sustaining glutamate (i.e., *PRODH)*, aspartate (i.e., *AST1*), alanine (i.e., *GPT*), asparagine (i.e., *ASNS*), and cysteine (i.e., *CTH*) levels suggest potential vulnerabilities involving these amino acids.

## Evidence Supporting NEAA Vulnerabilities in AML and CML

Various factors can lead to genetic alterations in cancers that result in the downregulation of an enzyme involved in the biosynthesis of a NEAA, thus rendering the cancer cells dependent on extracellular sources of the NEAA for proliferation and growth. Based on the expression changes observed in AML and CML samples described in the previous section, significant NEAA biosynthesis pathways identified will be reviewed to identify high-priority NEAA vulnerabilities in AML and CML.

### There Is Strong Clinical Evidence Supporting Vulnerabilities to Aspartate-Derived NEAAs in Leukemias

The best evidence supporting amino acid vulnerabilities for the treatment of leukemias is based on the efficacy of the chemotherapeutic L-asparaginase (58–63), which is a bacteria-derived enzyme that hydrolyzes asparagine into aspartate and ammonia (64, 65). The enzyme also possesses off-target residual glutaminase activity (66, 67) and several studies have indicated that both its asparaginase and glutaminase activity are required for antileukemic efficacy depending on the expression of ASNS by the cancer (68–72). Generally, asparaginase may be beneficial for malignancies expressing low or no ASNS activity (68, 70, 73, 74), and several studies have investigated the use of asparaginase in non-hematologic malignancies, such as pancreatic, ovarian, and breast cancers (75–79). Furthermore, there is substantial clinical evidence that including asparaginase during the treatment of AML results in better treatment outcomes (80–87). While few studies in CML patients are available to assess the clinical efficacy of asparaginase (88–91), preclinical studies indicate that asparaginase may also be beneficial for CML treatment (92, 93).

Similar to asparaginase, many human cancers, such as melanoma, lymphoma, glioma, and prostate cancer, have low or no detectable expression of ASS1 (94–97). Therefore, several approaches have been investigated for depleting arginine in various cancer types and have led to the development of two pharmacological agents: arginine deiminase derived from bacteria (98–100) and human arginase 1 (101–110). Both are currently undergoing clinical trials (NCT03449901, NCT04587830, NCT02709512 and NCT03455140) and encouraging clinical efficacy has been reported (107, 110–116), including for AML (112, 117).

In our analysis, enzymes involved in the biosynthesis of aspartate (i.e., AST/GOT) were downregulated in AML (**Figures 3A, B**), where aspartate serves as a precursor of asparagine and arginine (**Figure 1**). Interestingly, most human cells express low levels of the mitochondrial aspartate/glutamate SLC25A12 transporter, thereby depending on AST-dependent aspartate synthesis (118–120). Furthermore, levels of aspartate has been linked to cancer cell proliferation (118–122) and have been shown to provide a competitive growth advantage to cancer cells under hypoxia by contributing to the formation of oxaloacetate and enabling NADH recycling for glycolysis (119, 120, 123, 124). Aminooxyacetic acid (125, 126), hydrazinosuccinic acid (126–129), and iGOT1-01 (130) have been identified as AST inhibitors. While limited information is available regarding the potential clinical efficacy of targeting the aspartate biosynthesis pathway in cancer, *in vitro* studies using these inhibitors have demonstrated that they can decreases the proliferation of MDA-MB-231 breast adenocarcinoma cells (125), PaTu-8902 pancreatic cancer cells (125), PaTu-8902 pancreatic cancer cells (130), and DLD1 colon cancer cells (130).

### Restriction of Glutamate or Glutamine Metabolism *Via* Inhibitors of Glutaminase Are Effective Against Myelodysplastic Syndrome

As previously mentioned, glutaminase (GLS) controls the formation of glutamate (**Figure 1**) and is used for various biosynthetic purposes by cells, in addition to NEAA biosynthesis, including to generate TCA cycle intermediates, glutathione (GSH), NADPH, nucleotides, and fatty acids (131). Therefore, successful strategies targeting the role of glutamate in cancers have focused on restricting glutamine metabolism *via* glutaminase inhibition (132). Studies investigating the non-competitive allosteric GLS1 inhibitor CB-839 have demonstrated that blocking glutamine metabolism has anticancer activity against triple-negative breast cancer, lung adenocarcinoma, chondrosarcoma, lymphomas, esophageal squamous cell carcinoma, and hepatocellular carcinoma (132–138). Currently, there are 12 ongoing clinical trials evaluating the CB-839 in patients with colorectal cancer (NCT02861300 and NCT03263429), myelodysplastic syndrome (NCT03047993), advanced stage non-small cell lung cancer (NCT04250545 and NCT03831932), diffuse astrocytoma (NCT03528642), ovarian cancer (NCT03944902), refractory multiple myeloma (NCT03798678), advanced or metastatic solid tumors (NCT03965845), metastatic renal cell carcinoma (NCT03428217), malignancies with NF1, KEAP1/NRF2, or STK11/LKB1 mutations (NCT03872427), and non-squamous non-small-cell lung cancer (NCT04265534). Additionally, recent interim results from a phase Ib clinical study of CB-839 in combination with azacitidine in patients with advanced myelodysplastic syndrome demonstrate the possible potential of targeting glutamate sources in leukemias, where the regimen was safely tolerated and 70% of MDS patients achieved a complete response to therapy (139). In addition to CB-839, compound 968 (C968) is another small molecule inhibitor of GLS (140) that has been demonstrated to have anticancer activity in ovarian, brain, pancreatic, and breast cancer (140–142).

#### Restriction of Other NEAAs Derived From Glutamate Decrease Myeloid Leukemia Proliferation

The biosynthesis of serine and its derivatives were also identified as possible targets in AML and CML (**Figures 3E, F** and **Figure 5E**), and consistent with the gene expression analysis, substantial evidence supports that serine, cysteine, and glycine play important roles in cancers, including myeloid malignancies. One-carbon metabolism is required for the synthesis of proteins, lipids, and nucleic acids, with serine being the main source of one-carbon units for methylation reactions that occur through the generation of S-adenosylmethionine (SAM) (143). Glycine is also a major source of methyl groups for one-carbon pools and is required for the biosynthesis of GSH and purines (51), whereas cysteine contributes to redox control and ATP production as a carbon source for biomass and energy production (144). Furthermore, restriction of serine (145–148), glycine (145, 148, 149), or cysteine (150) can decrease cancer cell proliferation. Strategies suppressing PHGDH, SHMT, or cysteine levels *via* the enzyme cyst(e)inase have demonstrated encouraging results. For AML, there is evidence that serine restriction or PHGDH inhibition can attenuate cell proliferation and that *PHGDH* expression is prognostic of AML overall survival (151). In addition, cysteine has been demonstrated to be critical for the survival of leukemic stem cells (LSCs) in AML patients, whereas glycine deprivation has been demonstrated to suppress AML cell proliferation (152). For CML, similar to our analysis, several studies have shown that levels of glutamate, serine, which is a precursor of cysteine, and alanine are increased by CML (153–155). Consistent with these NEAAs playing an important role in CML, restriction of serine (156, 157) and its derivatives glycine (156), or cysteine (158, 159) decreases CML proliferation, supporting that targeting these NEAA pathways may be a potential target for CML.

In addition to serine, glycine, and cysteine, several studies support that inhibiting proline biosynthesis in melanoma and breast cancers can impede cell growth (160–162). Furthermore, PYCR1 inhibitors with anticancer activity have been identified to explore the role of proline biosynthesis in AML (163, 164). Nevertheless, limited information is available regarding the effect of inhibiting either enzymes involved in proline biosynthesis or directly restricting proline in AML. Rather AML studies available have indicated that proline uptake is elevated in LSCs isolated from *de novo* AML patients (165), and that the proline metabolism pathway is significantly impacted by differences in the oncogenic receptor tyrosine kinase FLT3 status of pediatric AML samples (166) or in AML cells overexpressing the proto-oncogene *EVI1 (* 167).

Alanine is secreted by skeletal muscles and provides the carbon source for hepatic gluconeogenesis (168, 169), yet limited information is available about the role of alanine metabolism in myeloid leukemias. Nevertheless, evidence supports that alanine provides a source of α-ketoglutarate for pancreatic and breast cancers that can be used to fuel the TCA cycle (170, 171) or remodel the extracellular matrix for metastasis (172). Additionally, KRAS mutations have been demonstrated to induce the expression of ALT2/GPT2 and drive α-ketoglutarate production and cell growth (173). Few studies have investigated pharmacological strategies for targeting alanine metabolism, yet l-cycloserine has been identified as an inhibitor of ALT/GPT that can attenuate the *in vitro* and *in vivo* growth of LLC1 Lewis lung carcinoma cells (173). Taken together, there is substantial evidence that the NEAA biosynthesis pathways dysregulated in AML and CML are promising targets for myeloid leukemias.

## GCN2 and mTOR Pathways can Modulate Resistance to Strategies Targeting NEAA Vulnerabilities

**Figure 6:**
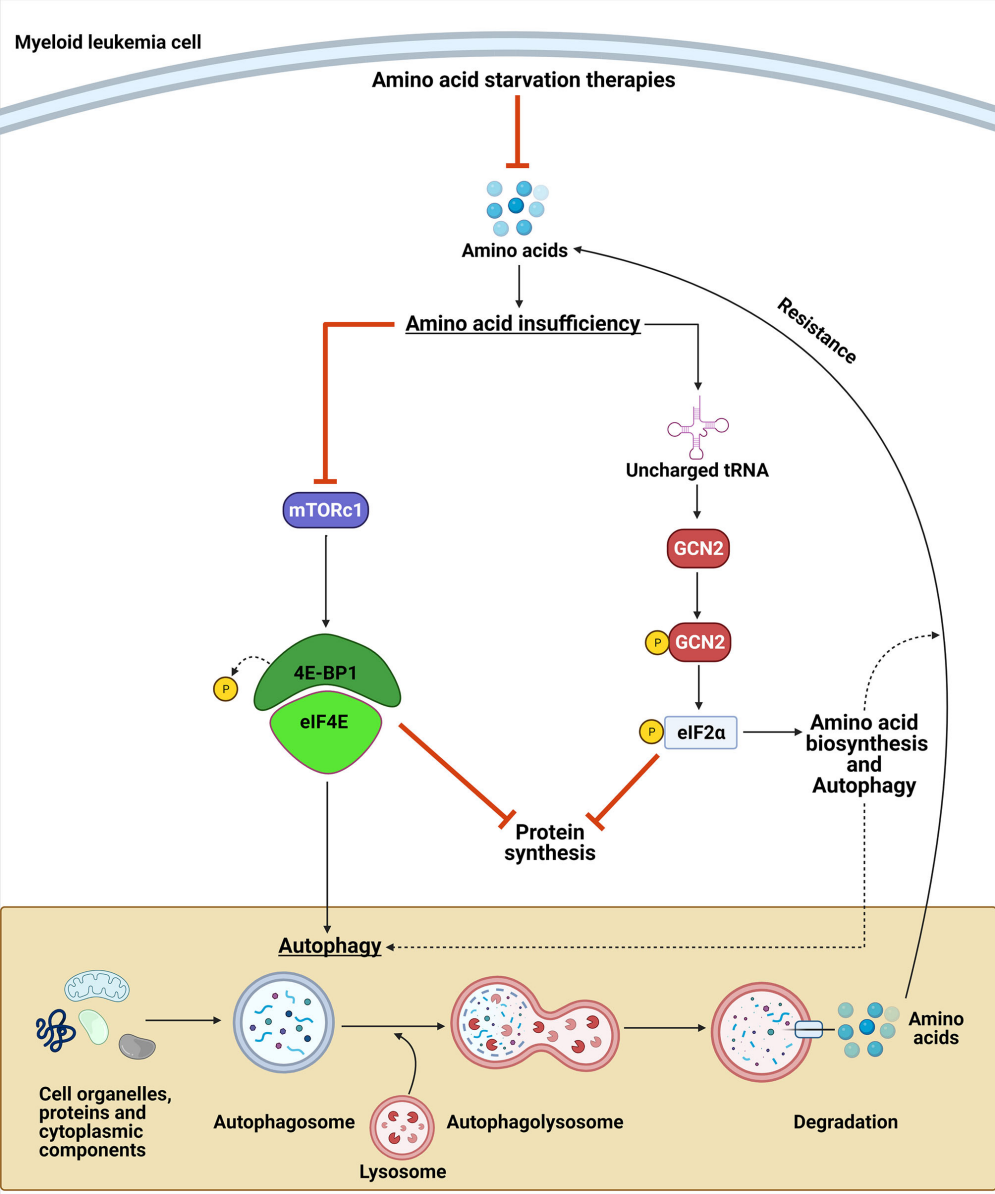
GCN2 and mTORC1 contribute to amino acid deprivation resistance. Upon amino acid deprivation, the accumulation of uncharged tRNA leads to the activation of the GCN2 kinase, which phosphorylates eIF2α. GCN2 activation can lead to a resistance to the amino acid restriction by inhibiting protein synthesis and by upregulating the translation of amino acid transporters, enzymes involved in amino acid biosynthesis, and proteins involved in autophagy. In contrast, amino acid suppression leads to inactivation of mTORC1, which induces autophagy and leads to a decrease in protein synthesis. Collectively, these mechanisms provide amino acid sources to maintain myeloid leukemia cell proliferation upon NEAA deprivation.

Strategies targeting NEAA biosynthesis vulnerabilities can be a promising approach for myeloid malignancies. However, cancer cell adaptation to nutrient stress can lead to drug resistance or treatment failure (174). This adaptation can be due to upregulation of amino acid transporters and/or cell changes leading to increased availability of NEAA pools (8, 174, 175). Therefore, understanding the mechanism by which myeloid leukemias adapt and sense NEAA restriction is important for optimizing therapeutic approaches targeting NEAA metabolic vulnerabilities. Two essential kinases that are involved in the adaptation to nutrient stress are mammalian target of rapamycin (mTOR) and general control nonderepressible-2 kinase (GCN2). Collectively, these pathways can decrease the cellular demand for amino acids while concurrently increasing their synthesis to overcome the nutrient stress and lead to resistance (**Figure 6**).

### Activation of the Amino Acid Response Pathway (AAR) Can Lead to Nutrient Stress Resistance

The GCN2 kinase activates the amino acid response (AAR) pathway by sensing amino acid insufficiency *via* direct binding to uncharged tRNAs that accumulate during nutrient stress (176). Upon activation *via* autophosphorylation, GCN2 phosphorylates its only known substrate, the α subunit of the translation eukaryotic initiation factor (eIF2) (177). Phosphorylation of eIF2α has two essential consequences that can affect cancer cell responses to amino acid deprivation (**Figure 6**). First, it attenuates the general protein translation of most mRNAs by blocking the activity of the eIF2B guanylate exchange factor, thereby limiting the availability of ternary complex required for initiating translation (178–180). Second, it activates the AAR pathway by concomitantly increasing the translation of a subset of mRNAs with upstream ORFs that regulate translation of the downstream main ORF (179, 181–183). The AAR pathway leads to the upregulation of amino acid transporters, aminoacyl-tRNA synthetases, enzymes involved in the biosynthesis of amino acids, and proteins involved in autophagy (29, 184–186).

In contrast to GCN2, which directly senses amino acid depletion, mTORC1 indirectly senses amino acid sufficiency through mechanisms that were recently elucidated (187–190). mTORC1 is a serine/threonine protein kinase that acts as an essential regulator of cell growth and metabolism in response to nutrient changes. SLC38A9, Sestrin1/2, and CASTOR1 have been identified as amino acid sensors upstream of mTORC1 that sense the availability of arginine and leucine (187–191). SLC38A9 stimulates mTORC1 activity through the regulation of Rag proteins, whereas Sestrin1/2 and CASTOR1 dissociate from GATOR2, which is a positive regulator of mTORC1, in the presence of leucine or arginine. Activated mTORC1 phosphorylates 4EBP1 and leads to the dissociation of 4EBP1 from EIF4E, enabling the formation of the translation initiation complex. In contrast, amino acid deficiency inactivates mTORC1 and suppresses 4EBP1 phosphorylation and protein synthesis (192–195). In addition to translation, mTORC1 is a negative regulator of autophagy, which is an intracellular process that allows orderly degradation and recycling of cellular components, including amino acids. Autophagy is regulated by mTORC1 through its phosphorylation of UNC-51-like kinase 1 (ULK) (196–200). Therefore, mTORC1 signaling plays a role in AA deprivation adaptation by limiting protein translation and increasing amino acid pools through autophagy (**Figure 6**).

Interestingly, there is substantial evidence supporting cross-talk between the GCN2/eIF2α pathway and mTORC1 signaling. Activating transcription factor 4 (ATF4) is induced by phosphorylation of eIF2α and several of its targets are mTORC1 inhibitors, including REDD1, GADD34, and Sestrin2 (201–203). Furthermore, autophagy regulation can be directly affected by ATF4 *via* the upregulation of many autophagy genes, including ATG16L, ATG12, ATG3, BECN1, LC3B, and p62 (185). Several studies have also demonstrated that inhibiting the GCN2/eIF2α pathway during amino acid deprivation prevents mTORC1 inactivation (204–206), whereas mTORC1 inhibition by rapamycin has been demonstrated to results in activation of the GCN2/eIF2α pathway (207).

Because mTORC1 and the GCN2/eIF2α pathway provide mechanisms of resistance to amino acid deprivation strategies, it is conceivable that blocking these adaptation pathways would further sensitize cancer cells to the nutrient stress. Consistent with that hypothesis, the development of a GCN2 inhibitor has been shown to enhance the antileukemic efficacy of L-asparaginase (208). Other studies have demonstrated similar adaptations and GCN2-dependent sensitizations (174, 209–215). While constitutive activation of mTORC1 can lead to resistance against targeted therapies (216), in the context of amino acid vulnerabilities, blocking the generation of amino acids from autophagic protein degradation can sensitize cancer cells to amino acid deprivation approaches (217). Consistent with this concept, blocking autophagy during asparagine (93, 218–222), arginine (106, 108, 223–229), and glutamine (230) restriction leads to enhanced efficacy.

## Conclusions and Future Perspectives

Cancers including myeloid leukemias use various energy sources to maintain their abnormal proliferation rate and therefore can become auxotrophic for particular NEAAs. Identifying these vulnerabilities in myeloid leukemias can therefore lead to new treatment options and improve survival rates. To date, L-asparaginase is the most successful clinical example of exploiting cancer nutrient dependencies. While outside of the scope of this review, there are several fuel sources aside from NEAA not considered here that contribute to metabolic vulnerabilities, including glucose, essential amino acids, fatty acids, lactate, and acetate (8–11). Furthermore, additional factors, including the expression of amino acid transporters (231–236) and the effect of stromal cells on the microenvironment, can contribute to cancer metabolism and proliferation (8). It is also clear that optimal treatment strategies will consider mechanisms of cell adaptation to nutrient stress. Our AML analysis identified that the biosynthesis of glutamate, aspartate, glutamine, proline, serine, asparagine, arginine, cysteine, and glycine are altered in TCGA samples, indicating that targeting multiple NEAAs may have a greater effect than a single agent approach. It is feasible that a combination approach to NEAA metabolic vulnerabilities may require lower drug exposures versus a single agent approach, and therefore can lead to better antileukemic efficacy while decreasing the risk of toxicities. Successful novel regimens with reduced risks of toxicities can lead to the development of new first-line therapy options for myeloid leukemia patients with limited treatment options.

## Footnotes

1. SEER*Explorer: An interactive website for SEER cancer statistics. Surveillance Research Program, National Cancer Institute. Available from https://seer.cancer.gov/explorer/. [Cited 2021 April 1]
2. https://www.ncbi.nlm.nih.gov/geo/query/acc.cgi?acc=GSE47927

## Author Contributions

CF and AB performed all data analysis. All authors contributed to the article and approved the submitted version.

## Funding

This study was supported by the University of Pittsburgh School of Pharmacy, the NIH TL1TR001858 Training Grant, the Rho Chi Society and American Foundation for Pharmaceutical Education, and the NIH Grant RO1 CA216815.

## Conflict of Interest

The authors declare that the research was conducted in the absence of any commercial or financial relationships that could be construed as a potential conflict of interest.

## Glossary

**Table:**

AA	Amino acid
AAR	Amino acid response
ALT1/GPT	Cytosolic alanine aminotransaminase 1/Glutamic pyruvic transaminase
ALT2/GPT2	Mitochondrial alanine transaminase 2/Glutamic pyruvic transaminase 2
ASL	Argininosuccinate lyase
ASNS	Asparagine synthetase
ASS1	Argininosuccinate synthase 1
AST1/GOT1	Aspartate Aminotransferase/glutamic-oxaloacetic transaminase 1
AST2/GOT2	Aspartate Aminotransferase/glutamic-oxaloacetic transaminase 2
ATF4	Activating transcription factor 4
BCAA	Branch-chain amino acid
BCAT1/2	Branched chain amino acid transaminase &frac12;
CBS	Cystathionine beta-synthase
CML	Chronic myeloid leukemia
CTH	Cystathionine gamma-lyase
eIF2α	Eukaryotic translation initiation factor 2 alpha
GCN2	General control nonderepressible-2
GLS/2	Glutaminase/2
GLUD1/2	Glutamate dehydrogenase &frac12;
GS/GLUL	Glutamine synthetase
GSH	Glutathione
HCT	Hematopoietic cell transplantations
HSC	Hematopoietic stem cell
LSC	Leukemic stem cell
mTOR	Mammalian target of rapamycin
NCCN	National Comprehensive Cancer Network
NEAA	Non-essential amino acid
OxPhos	Oxidative phosphorylation
3-PG	3-Phosphoglycerate
3-PHP	3-phosphohydroxypyruvate
3-PS	3-Phosphoserine
P5CDH/ALDH4A1	Pyrroline-5-carboxylate dehydrogenase/Aldehyde dehydrogenase 4 family member A1
P5CS/ALDH18A1	Pyrroline-5-carboxylate synthase/Aldehyde dehydrogenase 18 family member A1
PAH	Phenylalanine hydroxylase
PHGDH	Phosphoglycerate dehydrogenase
PRODH	Proline dehydrogenase 1
PSAT1	Phosphoserine aminotransferase 1
PSPH	Phosphoserine phosphatase
PYCR1	Pyrroline-5-carboxylate reductase 1
SAM	S-Adenosylmethionine
SHMT1/2	Serine hydroxymethyltransferase &frac12;
ULK	UNC-51-like kinase.

## References

[1] Siegel RL Miller KD Jemal A. Cancer Statistics, 2020. CA Cancer J Clin (2020) 70(1):7–30. 10.3322/caac.21590 31912902

[2] Shallis RM Wang R Davidoff A Ma X Zeidan AM. Epidemiology of Acute Myeloid Leukemia: Recent Progress and Enduring Challenges. Blood Rev (2019) 36:70–87. 10.1016/j.blre.2019.04.005 31101526

[3] DiNardo CD Jonas BA Pullarkat V Thirman MJ Garcia JS Wei AH. Azacitidine and Venetoclax in Previously Untreated Acute Myeloid Leukemia. N Engl J Med (2020) 383(7):617–29. 10.1056/NEJMoa2012971.Citedin:Pubmed 32786187

[4] Druker BJ Talpaz M Resta DJ Peng B Buchdunger E Ford JM. Efficacy and Safety of a Specific Inhibitor of the BCR-ABL Tyrosine Kinase in Chronic Myeloid Leukemia. N Engl J Med (2001) 344(14):1031–7. 10.1056/NEJM200104053441401 11287972

[5] Tanizawa A. Optimal Management for Pediatric Chronic Myeloid Leukemia. Pediatr Int (2016) 58(3):171–9. 10.1111/ped.12876 26646444

[6] Chan O Talati C Isenalumhe L Shams S Nodzon L Fradley M. Side-Effects Profile and Outcomes of Ponatinib in the Treatment of Chronic Myeloid Leukemia. Blood Adv (2020) 4(3):530–8. 10.1182/bloodadvances.2019000268 PMC7013263 32045474

[7] Issa GC Kantarjian HM Gonzalez GN Borthakur G Tang G Wierda W. Clonal Chromosomal Abnormalities Appearing in Philadelphia Chromosome–Negative Metaphases During CML Treatment. Blood (2017) 130(19):2084–91. 10.1182/blood-2017-07-792143%JBlood PMC5680612 28835440

[8] Vettore L Westbrook RL Tennant DA. New Aspects of Amino Acid Metabolism in Cancer. Br J Cancer (2020) 122(2):150–6. 10.1038/s41416-019-0620-5 PMC7052246 31819187

[9] Keenan MM Chi JT. Alternative Fuels for Cancer Cells. Cancer J (2015) 21(2):49–55. 10.1097/ppo.0000000000000104 25815843 PMC4380238

[10] Vazquez A Kamphorst JJ Markert EK Schug ZT Tardito S Gottlieb E. Cancer Metabolism at a Glance. J Cell Sci (2016) 129(18):3367–73. 10.1242/jcs.181016 PMC6518336 27635066

[11] Pavlova NN Thompson CB. The Emerging Hallmarks of Cancer Metabolism. Cell Metab (2016) 23(1):27–47. 10.1016/j.cmet.2015.12.006 26771115 PMC4715268

[12] Otten JJ Hellwig JP Meyers LD eds. Medicine Io. Dietary Reference Intakes: The Essential Guide to Nutrient Requirements Vol. 1344. Washington, DC: The National Academies Press (2006). Available at: https://www.nap.edu/catalog/11537/dietary-reference-intakes-the-essential-guide-to-nutrient-requirements.

[13] Fung MKL Chan GC. Drug-Induced Amino Acid Deprivation as Strategy for Cancer Therapy. J Hematol Oncol (2017) 10(1):144. 10.1186/s13045-017-0509-9 28750681 PMC5530962

[14] Hanahan D Weinberg Robert A. Hallmarks of Cancer: The Next Generation. Cell (2011) 144(5):646–74. 10.1016/j.cell.2011.02.013 21376230

[15] Lopez MJ MS. Biochemistry, Essential Amino Acids (2021). StatPearls Publishing. Available at: https://www.ncbi.nlm.nih.gov/books/NBK557845/?report=classic (Accessed January 2021). 32496725

[16] Morris CR Hamilton-Reeves J Martindale RG Sarav M Ochoa Gautier JB. Acquired Amino Acid Deficiencies: A Focus on Arginine and Glutamine. Nutr Clin Pract (2017) 32(1S):30S–47S. 10.1177/0884533617691250 28388380

[17] Nilsson A Haanstra JR Engqvist M Gerding A Bakker BM Klingmüller U. Quantitative Analysis of Amino Acid Metabolism in Liver Cancer Links Glutamate Excretion to Nucleotide Synthesis. PNAS (2020) 117(19):10294–304. 10.1073/pnas.1919250117 PMC7229649 32341162

[18] Asantewaa G Harris IS. Glutathione and its Precursors in Cancer. Curr Opin Biotechnol (2021) 68:292–9. 10.1016/j.copbio.2021.03.001 33819793

[19] Green CR Wallace M Divakaruni AS Phillips SA Murphy AN Ciaraldi TP. Branched-Chain Amino Acid Catabolism Fuels Adipocyte Differentiation and Lipogenesis. Nat Chem Biol (2016) 12(1):15–21. 10.1038/nchembio.1961 26571352 PMC4684771

[20] Sugden MC Watts DI West PS Norman Palmer T. Proline and Hepatic Lipogenesis. Biochim Biophys Acta (BBA) - Gen Subj (1984) 798(3):368–73. 10.1016/0304-4165(84)90111-9 6712996

[21] Corbet C Feron O. Cancer Cell Metabolism and Mitochondria: Nutrient Plasticity for TCA Cycle Fueling. Biochim Biophys Acta (BBA) - Rev Cancer (2017) 1868(1):7–15. 10.1016/j.bbcan.2017.01.002 28110019

[22] Renner K Singer K Koehl GE Geissler EK Peter K Siska PJ. Metabolic Hallmarks of Tumor and Immune Cells in the Tumor Microenvironment [Review]. English (2017) 8:248. 10.3389/fimmu.2017.00248 PMC5340776 28337200

[23] Nakanishi S Cleveland JL. Polyamine Homeostasis in Development and Disease. Med Sci (Basel) (2021) 9(2):28. 10.3390/medsci9020028 34068137 PMC8162569

[24] Pasini A Caldarera CM Giordano E. Chromatin Remodeling by Polyamines and Polyamine Analogs. Amino Acids (2014) 46(3):595–603. 10.1007/s00726-013-1550-9 23836422

[25] Shyh-Chang N Locasale JW Lyssiotis CA Zheng Y Teo RY Ratanasirintrawoot S. Influence of Threonine Metabolism on S-Adenosylmethionine and Histone Methylation. Science (2013) 339(6116):222–6. 10.1126/science.1226603%JScience PMC3652341 23118012

[26] Sun L Zhang H Gao P. Metabolic Reprogramming and Epigenetic Modifications on the Path to Cancer. Protein Cell (2021). 10.1007/s13238-021-00846-7 PMC9243210 34050894

[27] Maddocks ODK Labuschagne CF Adams PD Vousden KH. Serine Metabolism Supports the Methionine Cycle and DNA/RNA Methylation Through De Novo ATP Synthesis in Cancer Cells. Mol Cell (2016) 61(2):210–21. 10.1016/j.molcel.2015.12.014 PMC4728077 26774282

[28] Ulrey CL Liu L Andrews LG Tollefsbol TO. The Impact of Metabolism on DNA Methylation. Hum Mol Genet (2005) 14(suppl_1):R139–47. 10.1093/hmg/ddi100%JHumanMolecularGenetics 15809266

[29] Pathria G Ronai ZA. Harnessing the Co-Vulnerabilities of Amino Acid-Restricted Cancers. Cell Metab (2021) 33(1):9–20. 10.1016/j.cmet.2020.12.009 33406406 PMC7837405

[30] Maggi M Scotti C. Enzymes in Metabolic Anticancer Therapy. Adv Exp Med Biol (2019) 1148:173–99. 10.1007/978-981-13-7709-9_9 31482500

[31] Tabe Y Lorenzi PL Konopleva M. Amino Acid Metabolism in Hematologic Malignancies and the Era of Targeted Therapy. Blood (2019) 134(13):1014–23. 10.1182/blood.2019001034 PMC6764269 31416801

[32] Boyko M Gruenbaum SE Gruenbaum BF Shapira Y Zlotnik A. Brain to Blood Glutamate Scavenging as a Novel Therapeutic Modality: A Review. J Neural Transm (Vienna) (2014) 121(8):971–9. 10.1007/s00702-014-1181-7 PMC4382077 24623040

[33] Mastorodemos V Kotzamani D Zaganas I Arianoglou G Latsoudis H Plaitakis A. Human GLUD1 and GLUD2 Glutamate Dehydrogenase Localize to Mitochondria and Endoplasmic Reticulum. Biochem Cell Biol (2009) 87(3):505–16. 10.1139/o09-008 19448744

[34] Doebbe A Keck M La Russa M Mussgnug JH Hankamer B Tekce E. The Interplay of Proton, Electron, and Metabolite Supply for Photosynthetic H2 Production in Chlamydomonas Reinhardtii. J Biol Chem (2010) 285(39):30247–60. 10.1074/jbc.M110.122812 PMC2943295 20581114

[35] Carr EL Kelman A Wu GS Gopaul R Senkevitch E Aghvanyan A. Glutamine Uptake and Metabolism are Coordinately Regulated by ERK/MAPK During T Lymphocyte Activation. J Immunol (2010) 185(2):1037–44. 10.4049/jimmunol.0903586 PMC2897897 20554958

[36] Rajagopalan KN DeBerardinis RJ. Role of Glutamine in Cancer: Therapeutic and Imaging Implications. J Nucl Med (2011) 52(7):1005–8. 10.2967/jnumed.110.084244 PMC3337771 21680688

[37] Perez-Arellano I Carmona-Alvarez F Martinez AI Rodriguez-Diaz J Cervera J. Pyrroline-5-Carboxylate Synthase and Proline Biosynthesis: From Osmotolerance to Rare Metabolic Disease. Protein Sci (2010) 19(3):372–82. 10.1002/pro.340 PMC2866264 20091669

[38] Walsh DA Sallach HJ. Comparative Studies on the Pathways for Serine Biosynthesis in Animal Tissues. J Biol Chem (1966) 241(17):4068–76. 10.1016/S0021-9258(18)99812-1 5920812

[39] Chiu M Taurino G Bianchi MG Kilberg MS Bussolati O. Asparagine Synthetase in Cancer: Beyond Acute Lymphoblastic Leukemia. Front Oncol (2019) 9:1480. 10.3389/fonc.2019.01480 31998641 PMC6962308

[40] Keshet R Szlosarek P Carracedo A Erez A. Rewiring Urea Cycle Metabolism in Cancer to Support Anabolism. Nat Rev Cancer (2018) 18(10):634–45. 10.1038/s41568-018-0054-z 30194362

[41] Belalcazar AD Ball JG Frost LM Valentovic MA Wilkinson JT. Transsulfuration Is a Significant Source of Sulfur for Glutathione Production in Human Mammary Epithelial Cells. ISRN Biochem (2014) 2013:637897. 10.1155/2013/637897 24634789 PMC3949734

[42] Leikam C Hufnagel A Walz S Kneitz S Fekete A Muller MJ. Cystathionase Mediates Senescence Evasion in Melanocytes and Melanoma Cells. Oncogene (2014) 33(6):771–82. 10.1038/onc.2012.641 23353821

[43] Lien EC Ghisolfi L Geck RC Asara JM Toker A. Oncogenic PI3K Promotes Methionine Dependency in Breast Cancer Cells Through the Cystine-Glutamate Antiporter xCT. Sci Signal (2017) 10(510). 10.1126/scisignal.aao6604.Citedin:Pubmed PMC5808948 29259101

[44] Bannai S. Transport of Cystine and Cysteine in Mammalian Cells. Biochim Biophys Acta (1984) 779(3):289–306. 10.1016/0304-4157(84)90014-5 6383474

[45] Iglehart JK York RM Modest AP Lazarus H Livingston DM. Cystine Requirement of Continuous Human Lymphoid Cell Lines of Normal and Leukemic Origin. J Biol Chem (1977) 252(20):7184–91. 10.1016/S0021-9258(19)66953-X 903356

[46] Uren JR Lazarus H. L-Cyst(E)Ine Requirements of Malignant Cells and Progress Toward Depletion Therapy. Cancer Treat Rep (1979) 63(6):1073–9. 466647

[47] McBean GJ. The Transsulfuration Pathway: A Source of Cysteine for Glutathione in Astrocytes. Amino Acids (2012) 42(1):199–205. 10.1007/s00726-011-0864-8 21369939

[48] Sbodio JI Snyder SH Paul BD. Regulators of the Transsulfuration Pathway. Br J Pharmacol (2019) 176(4):583–93. 10.1111/bph.14446 PMC6346075 30007014

[49] Stipanuk MH Dominy JE Jr. Lee JI Coloso RM. Mammalian Cysteine Metabolism: New Insights Into Regulation of Cysteine Metabolism. J Nutr (2006) 136(6 Suppl):1652S–9S. 10.1093/jn/136.6.1652S 16702335

[50] Liu N Lin X Huang C. Activation of the Reverse Transsulfuration Pathway Through NRF2/CBS Confers Erastin-Induced Ferroptosis Resistance. Br J Cancer (2020) 122(2):279–92. 10.1038/s41416-019-0660-x PMC7052275 31819185

[51] Amelio I Cutruzzola F Antonov A Agostini M Melino G. Serine and Glycine Metabolism in Cancer. Trends Biochem Sci (2014) 39(4):191–8. 10.1016/j.tibs.2014.02.004 PMC3989988 24657017

[52] Garrow TA Brenner AA Whitehead VM Chen XN Duncan RG Korenberg JR. Cloning of Human cDNAs Encoding Mitochondrial and Cytosolic Serine Hydroxymethyltransferases and Chromosomal Localization. J Biol Chem (1993) 268(16):11910–6. 10.1016/S0021-9258(19)50286-1 8505317

[53] Fitzpatrick PF. Tetrahydropterin-Dependent Amino Acid Hydroxylases. Annu Rev Biochem (1999) 68:355–81. 10.1146/annurev.biochem.68.1.355 10872454

[54] Tang Z Li C Kang B Gao G Li C Zhang Z. GEPIA: A Web Server for Cancer and Normal Gene Expression Profiling and Interactive Analyses. Nucleic Acids Res (2017) 45(W1):W98–W102. 10.1093/nar/gkx247 28407145 PMC5570223

[55] Tang Z Kang B Li C Chen T Zhang Z. GEPIA2: An Enhanced Web Server for Large-Scale Expression Profiling and Interactive Analysis. Nucleic Acids Res (2019) 47(W1):W556–w560. 10.1093/nar/gkz430.Citedin:Pubmed 31114875 PMC6602440

[56] Goldman M Craft B Hastie M Repečka K McDade F Kamath A. The UCSC Xena Platform for Public and Private Cancer Genomics Data Visualization and Interpretation. bioRxiv (2019) 326470. 10.1101/326470%JbioRxiv

[57] Goldman MJ Craft B Hastie M Repečka K McDade F Kamath A. Visualizing and Interpreting Cancer Genomics Data *via* the Xena Platform. Nat Biotechnol (2020) 38(6):675–8. 10.1038/s41587-020-0546-8 PMC7386072 32444850

[58] Sallan SE Hitchcock-Bryan S Gelber R Cassady JR Frei E Nathan DG. Influence of Intensive Asparaginase in the Treatment of Childhood Non-T-Cell Acute Lymphoblastic Leukemia. Can Res (1983) 43:5601–7. 6352020

[59] Amylon MD Shuster J Pullen J Berard C Link MP Wharam M. Intensive High-Dose Asparaginase Consolidation Improves Survival for Pediatric Patients With T Cell Acute Lymphoblastic Leukemia and Advanced Stage Lymphoblastic Lymphoma: A Pediatric Oncology Group Study. Leukemia (1999) 13(3):335–42. 10.1038/sj.leu.2401310 10086723

[60] Silverman LB Gelber RD Dalton VK Asselin BL Barr RD Clavell LA. Improved Outcome for Children With Acute Lymphoblastic Leukemia: Results of Dana-Farber Consortium Protocol 91-01. Blood (2001) 97(5):1211–8. 10.1182/blood.V97.5.1211 11222362

[61] Pui CH Campana D Pei D Bowman WP Sandlund JT Kaste SC. Treating Childhood Acute Lymphoblastic Leukemia Without Cranial Irradiation. N Engl J Med (2009) 360(26):2730–41. 10.1056/NEJMoa0900386 PMC2754320 19553647

[62] Ertel IJ Nesbit ME Hammond D Weiner J Sather H. Effective Dose of L-Asparaginase for Induction of Remission in Previously Treated Children With Acute Lymphocytic Leukemia: A Report From Childrens Cancer Study Group. Can Res (1979) 39:3893–6. 383278

[63] Clavell LA Gelber RD Cohen HJ Hitchcock-Bryan S Cassady JR Tarbell NJ. Four-Agent Induction and Intensive Asparaginase Therapy for Treatment of Childhood Acute Lymphoblastic Leukemia. N Engl J Med (1986) 315:657–63. 10.1056/NEJM198609113151101 2943992

[64] Fernandez CA Cai X Elozory A Liu C Panetta JC Jeha S. High-Throughput Asparaginase Activity Assay in Serum of Children With Leukemia. Int J Clin Exp Med (2013) 6(7):478–87. PMC3731178 23936585

[65] Broome JD. L-Asparaginase: Discovery and Development as a Tumor-Inhibitory Agent. Cancer Treat Rep (1981) 65 Suppl 4:111–4. 7049374

[66] Miller HK Balis ME. Glutaminase Activity of L-Asparagine Amidohydrolase. Biochem Pharmacol (1969) 18(9):2225–32. 10.1016/0006-2952(69)90329-3 4899451

[67] Distasio JA Salazar AM Nadji M Durden DL. Glutaminase-Free Asparaginase From Vibrio Succinogenes: An Antilymphoma Enzyme Lacking Hepatotoxicity. Int J Cancer (1982) 30(3):343–7. 10.1002/ijc.2910300314 6752048

[68] Chan WK Horvath TD Tan L Link T Harutyunyan KG Pontikos MA. Glutaminase Activity of L-Asparaginase Contributes to Durable Preclinical Activity Against Acute Lymphoblastic Leukemia. Mol Cancer Ther (2019) 18(9):1587–92. 10.1158/1535-7163.MCT-18-1329 PMC6726508 31209181

[69] Wu MC Arimura GK Yunis AA. Mechanism of Sensitivity of Cultured Pancreatic Carcinoma to Asparaginase. Int J Cancer (1978) 22(6):728–33. 10.1002/ijc.2910220615 363626

[70] Chan WK Lorenzi PL Anishkin A Purwaha P Rogers DM Sukharev S. The Glutaminase Activity of L-Asparaginase is Not Required for Anticancer Activity Against ASNS-Negative Cells. Blood (2014) 123(23):3596–606. 10.1182/blood-2013-10-535112 PMC4047499 24659632

[71] Offman MN Krol M Patel N Krishnan S Liu J Saha V. Rational Engineering of L-Asparaginase Reveals Importance of Dual Activity for Cancer Cell Toxicity. Blood (2011) 117(5):1614–21. 10.1182/blood-2010-07-298422 21106986

[72] Parmentier JH Maggi M Tarasco E Scotti C Avramis VI Mittelman SD. Glutaminase Activity Determines Cytotoxicity of L-Asparaginases on Most Leukemia Cell Lines. Leuk Res (2015) 39(7):757–62. 10.1016/j.leukres.2015.04.008 PMC4458142 25941002

[73] Kiriyama Y Kubota M Takimoto T Kitoh T Tanizawa A Akiyama Y. Biochemical Characterization of U937 Cells Resistant to L-Asparaginase: The Role of Asparagine Synthetase. Leukemia (1989) 3(4):294–7. 2564453

[74] Stams WA den Boer ML Beverloo HB Meijerink JP Stigter RL van Wering ER. Sensitivity to L-Asparaginase is Not Associated With Expression Levels of Asparagine Synthetase in T(12;21)+ Pediatric ALL. Blood (2003) 101(7):2743–7. 10.1182/blood-2002-08-2446 12433682

[75] Yang H He X Zheng Y Feng W Xia X Yu X. Down-Regulation of Asparagine Synthetase Induces Cell Cycle Arrest and Inhibits Cell Proliferation of Breast Cancer. Chem Biol Drug Des (2014) 84(5):578–84. 10.1111/cbdd.12348 24775638

[76] Domenech C Thomas X Chabaud S Baruchel A Gueyffier F Mazingue F. L-Asparaginase Loaded Red Blood Cells in Refractory or Relapsing Acute Lymphoblastic Leukaemia in Children and Adults: Results of the GRASPALL 2005-01 Randomized Trial. Br J Haematol (2011) 153(1):58–65. 10.1111/j.1365-2141.2011.08588.x 21332712

[77] Hammel P Fabienne P Mineur L Metges JP Andre T de la Fouchardiere C. Erythrocyte-Encapsulated Asparaginase (Eryaspase) Combined With Chemotherapy in Second-Line Treatment of Advanced Pancreatic Cancer: An Open-Label, Randomized Phase IIb Trial. Eur J Cancer (2020) 124:91–101. 10.1016/j.ejca.2019.10.020 31760314

[78] Lorenzi PL Reinhold WC Rudelius M Gunsior M Shankavaram U Bussey KJ. Asparagine Synthetase as a Causal, Predictive Biomarker for L-Asparaginase Activity in Ovarian Cancer Cells. Mol Cancer Ther (2006) 5(11):2613–23. 10.1158/1535-7163.MCT-06-0447 17088436

[79] Lorenzi PL Llamas J Gunsior M Ozbun L Reinhold WC Varma S. Asparagine Synthetase is a Predictive Biomarker of L-Asparaginase Activity in Ovarian Cancer Cell Lines. Mol Cancer Ther (2008) 7(10):3123–8. 10.1158/1535-7163.MCT-08-0589 PMC4123961 18852115

[80] Capizzi RL Poole M Cooper MR Richards F 2nd Stuart JJ Jackson DV Jr.. Treatment of Poor Risk Acute Leukemia With Sequential High-Dose ARA-C and Asparaginase. Blood (1984) 63(3):694–700. 6696996

[81] Capizzi RL Davis R Powell B Cuttner J Ellison RR Cooper MR. Synergy Between High-Dose Cytarabine and Asparaginase in the Treatment of Adults With Refractory and Relapsed Acute Myelogenous Leukemia–a Cancer and Leukemia Group B Study. J Clin Oncol (1988) 6(3):499–508. 10.1200/JCO.1988.6.3.499 3162515

[82] Wells RJ Woods WG Lampkin BC Nesbit ME Lee JW Buckley JD. Impact of High-Dose Cytarabine and Asparaginase Intensification on Childhood Acute Myeloid Leukemia: A Report From the Childrens Cancer Group. J Clin Oncol (1993) 11(3):538–45. 10.1200/JCO.1993.11.3.538 8445429

[83] Horikoshi A Takei K Iriyama N Uenogawa K Ishizuka H Shiraiwa H. Effect of L-Asparaginase Combined With Vincristine and Prednisolone on Acute Myeloblastic Leukemia (M0) Associated With non-Hodgkin Lymphoma. Acta Haematol (2009) 122(1):54–7. 10.1159/000243725 19816010

[84] Buaboonnam J Cao X Pauley JL Pui CH Ribeiro RC Rubnitz JE. Sequential Administration of Methotrexate and Asparaginase in Relapsed or Refractory Pediatric Acute Myeloid Leukemia. Pediatr Blood Cancer (2013) 60(7):1161–4. 10.1002/pbc.24470 PMC4005561 23335430

[85] Ahmed T Holwerda S Klepin HD Isom S Ellis LR Lyerly S. High Dose Cytarabine, Mitoxantrone and L-Asparaginase (HAMA) Salvage for Relapsed or Refractory Acute Myeloid Leukemia (AML) in the Elderly. Leuk Res (2015) 39(9):945–9. 10.1016/j.leukres.2015.05.010 PMC4546894 26154683

[86] Emadi A Zokaee H Sausville EA. Asparaginase in the Treatment of non-ALL Hematologic Malignancies. Cancer Chemother Pharmacol (2014) 73(5):875–83. 10.1007/s00280-014-2402-3 24515335

[87] Ohnuma T Holland JF Nagel G Arneault GS. Effects of L-Asparaginase in Acute Myelocytic Leukemia. JAMA (1969) 210(10):1919–21. 10.1001/jama.210.10.1919 5260766

[88] Wadhwa J Szydlo RM Apperley JF Chase A Bua M Marin D. Factors Affecting Duration of Survival After Onset of Blastic Transformation of Chronic Myeloid Leukemia. Blood (2002) 99(7):2304–9. 10.1182/blood.v99.7.2304 11895760

[89] Naqvi K Cortes JE Luthra R O’Brien S Wierda W Borthakur G. Characteristics and Outcome of Chronic Myeloid Leukemia Patients With E255K/V BCR-ABL Kinase Domain Mutations. Int J Hematol (2018) 107(6):689–95. 10.1007/s12185-018-2422-6 29464484

[90] Meyran D Petit A Guilhot J Suttorp M Sedlacek P De Bont E. Lymphoblastic Predominance of Blastic Phase in Children With Chronic Myeloid Leukaemia Treated With Imatinib: A Report From the I-CML-Ped Study. Eur J Cancer (2020) 137:224–34. 10.1016/j.ejca.2020.06.024 32799036

[91] Morita K Jabbour E Ravandi F Borthakur G Khoury JD Hu S. Clinical Outcomes of Patients With Chronic Myeloid Leukemia With Concurrent Core Binding Factor Rearrangement and Philadelphia Chromosome. Clin Lymphoma Myeloma Leuk (2021) 21(5):338–44. 10.1016/j.clml.2020.12.025.Citedin:Pubmed PMC11513896 33597098

[92] Spiro TE Mattelaer MA Efira A Stryckmans P. Sensitivity of Myeloid Progenitor Cells in Healthy Subjects and Patients With Chronic Myeloid Leukemia to Chemotherapeutic Agents. J Natl Cancer Inst (1981) 66(6):1053–9. 10.1093/jnci/66.6.1053 6941040

[93] Song P Ye L Fan J Li Y Zeng X Wang Z. Asparaginase Induces Apoptosis and Cytoprotective Autophagy in Chronic Myeloid Leukemia Cells. Oncotarget (2015) 6(6):3861–73. 10.18632/oncotarget.2869 PMC4414159 25738356

[94] Mussai F Egan S Higginbotham-Jones J Perry T Beggs A Odintsova E. Arginine Dependence of Acute Myeloid Leukemia Blast Proliferation: A Novel Therapeutic Target. Blood (2015) 125(15):2386–96. 10.1182/blood-2014-09-600643 PMC4416943 25710880

[95] Sugimura K Ohno T Kusuyama T Azuma I. High Sensitivity of Human Melanoma Cell Lines to the Growth Inhibitory Activity of Mycoplasmal Arginine Deiminase *In Vitro* . Melanoma Res (1992) 2(3):191–6. 10.1097/00008390-199209000-00007 1450673

[96] Wheatley DN. Arginine Deprivation and Metabolomics: Important Aspects of Intermediary Metabolism in Relation to the Differential Sensitivity of Normal and Tumour Cells. Semin Cancer Biol (2005) 15(4):247–53. 10.1016/j.semcancer.2005.04.002 15886013

[97] Wheatley DN Kilfeather R Stitt A Campbell E. Integrity and Stability of the Citrulline-Arginine Pathway in Normal and Tumour Cell Lines. Cancer Lett (2005) 227(2):141–52. 10.1016/j.canlet.2005.01.004 16112417

[98] Takaku H Takase M Abe S Hayashi H Miyazaki K. *In Vivo* Anti-Tumor Activity of Arginine Deiminase Purified From Mycoplasma Arginini. Int J Cancer (1992) 51(2):244–9. 10.1002/ijc.2910510213 1568792

[99] Sugimura K Fukuda S Wada Y Taniai M Suzuki M Kimura T. Identification and Purification of Arginine Deiminase That Originated From Mycoplasma Arginini. Infect Immun (1990) 58(8):2510–5. 10.1128/IAI.58.8.2510-2515.1990 PMC258848 2370103

[100] Izzo F Marra P Beneduce G Castello G Vallone P De Rosa V. Pegylated Arginine Deiminase Treatment of Patients With Unresectable Hepatocellular Carcinoma: Results From Phase I/II Studies. J Clin Oncol (2004) 22(10):1815–22. 10.1200/JCO.2004.11.120 15143074

[101] Cheng PN Leung YC Lo WH Tsui SM Lam KC. Remission of Hepatocellular Carcinoma With Arginine Depletion Induced by Systemic Release of Endogenous Hepatic Arginase Due to Transhepatic Arterial Embolisation, Augmented by High-Dose Insulin: Arginase as a Potential Drug Candidate for Hepatocellular Carcinoma. Cancer Lett (2005) 224(1):67–80. 10.1016/j.canlet.2004.10.050 15911102

[102] Storr JM Burton AF. The Effects of Arginine Deficiency on Lymphoma Cells. Br J Cancer (1974) 30(1):50–9. 10.1038/bjc.1974.112 PMC2009188 4528778

[103] Savoca KV Davis FF van Es T McCoy JR Palczuk NC. Cancer Therapy With Chemically Modified Enzymes. II. The Therapeutic Effectiveness of Arginase, and Arginase Modified by the Covalent Attachment of Polyethylene Glycol, on the Taper Liver Tumor and the L5178Y Murine Leukemia. Cancer Biochem Biophys (1984) 7(3):261–8. 6488153

[104] Cheng PN Lam TL Lam WM Tsui SM Cheng AW Lo WH. Pegylated Recombinant Human Arginase (Rharg-Peg5,000mw) Inhibits the *In Vitro* and *In Vivo* Proliferation of Human Hepatocellular Carcinoma Through Arginine Depletion. Cancer Res (2007) 67(1):309–17. 10.1158/0008-5472.CAN-06-1945 17210712

[105] Lam TL Wong GK Chow HY Chong HC Chow TL Kwok SY. Recombinant Human Arginase Inhibits the *In Vitro* and *In Vivo* Proliferation of Human Melanoma by Inducing Cell Cycle Arrest and Apoptosis. Pigment Cell Melanoma Res (2011) 24(2):366–76. 10.1111/j.1755-148X.2010.00798.x 21029397

[106] Zeng X Li Y Fan J Zhao H Xian Z Sun Y. Recombinant Human Arginase Induced Caspase-Dependent Apoptosis and Autophagy in non-Hodgkin’s Lymphoma Cells. Cell Death Dis (2013) 4:e840. 10.1038/cddis.2013.359 24113174 PMC3824669

[107] Yau T Cheng PN Chan P Chen L Yuen J Pang R. Preliminary Efficacy, Safety, Pharmacokinetics, Pharmacodynamics and Quality of Life Study of Pegylated Recombinant Human Arginase 1 in Patients With Advanced Hepatocellular Carcinoma. Invest New Drugs (2015) 33(2):496–504. 10.1007/s10637-014-0200-8 25666409

[108] Nasreddine G El-Sibai M Abi-Habib RJ. Cytotoxicity of [HuArgI (Co)-PEG5000]-Induced Arginine Deprivation to Ovarian Cancer Cells is Autophagy Dependent. Invest New Drugs (2020) 38(1):10–9. 10.1007/s10637-019-00756-w 30887252

[109] Hsueh EC Knebel SM Lo WH Leung YC Cheng PN Hsueh CT. Deprivation of Arginine by Recombinant Human Arginase in Prostate Cancer Cells. J Hematol Oncol (2012) 5:17. 10.1186/1756-8722-5-17 22546217 PMC3403903

[110] Yau T Cheng PN Chan P Chan W Chen L Yuen J. A Phase 1 Dose-Escalating Study of Pegylated Recombinant Human Arginase 1 (Peg-Rharg1) in Patients With Advanced Hepatocellular Carcinoma. Invest New Drugs (2013) 31(1):99–107. 10.1007/s10637-012-9807-9 22426640 PMC3553413

[111] Feun LG Marini A Walker G Elgart G Moffat F Rodgers SE. Negative Argininosuccinate Synthetase Expression in Melanoma Tumours may Predict Clinical Benefit From Arginine-Depleting Therapy With Pegylated Arginine Deiminase. Br J Cancer (2012) 106(9):1481–5. 10.1038/bjc.2012.106 PMC3341859 22472884

[112] Tsai HJ Jiang SS Hung WC Borthakur G Lin SF Pemmaraju N. A Phase II Study of Arginine Deiminase (ADI-PEG20) in Relapsed/Refractory or Poor-Risk Acute Myeloid Leukemia Patients. Sci Rep (2017) 7(1):11253. 10.1038/s41598-017-10542-4 28900115 PMC5595917

[113] Ott PA Carvajal RD Pandit-Taskar N Jungbluth AA Hoffman EW Wu BW. Phase I/II Study of Pegylated Arginine Deiminase (ADI-PEG 20) in Patients With Advanced Melanoma. Invest New Drugs (2013) 31(2):425–34. 10.1007/s10637-012-9862-2 PMC4169197 22864522

[114] Glazer ES Piccirillo M Albino V Di Giacomo R Palaia R Mastro AA. Phase II Study of Pegylated Arginine Deiminase for Nonresectable and Metastatic Hepatocellular Carcinoma. J Clin Oncol (2010) 28(13):2220–6. 10.1200/JCO.2009.26.7765 20351325

[115] De Santo C Cheng P Beggs A Egan S Bessudo A Mussai F. Metabolic Therapy With PEG-Arginase Induces a Sustained Complete Remission in Immunotherapy-Resistant Melanoma. J Hematol Oncol (2018) 11(1):68. 10.1186/s13045-018-0612-6 29776373 PMC5960181

[116] Curley SA Bomalaski JS Ensor CM Holtsberg FW Clark MA. Regression of Hepatocellular Cancer in a Patient Treated With Arginine Deiminase. Hepatogastroenterology (2003) 50(53):1214–6. 14571701

[117] Tanios R Bekdash A Kassab E Stone E Georgiou G Frankel AE. Human Recombinant Arginase I(Co)-PEG5000 [HuArgI(Co)-PEG5000]-Induced Arginine Depletion is Selectively Cytotoxic to Human Acute Myeloid Leukemia Cells. Leuk Res (2013) 37(11):1565–71. 10.1016/j.leukres.2013.08.007 24018014

[118] Birsoy K Wang T Chen WW Freinkman E Abu-Remaileh M Sabatini DM. An Essential Role of the Mitochondrial Electron Transport Chain in Cell Proliferation Is to Enable Aspartate Synthesis. Cell (2015) 162(3):540–51. 10.1016/j.cell.2015.07.016 PMC4522279 26232224

[119] Garcia-Bermudez J Baudrier L La K Zhu XG Fidelin J Sviderskiy VO. Aspartate is a Limiting Metabolite for Cancer Cell Proliferation Under Hypoxia and in Tumours. Nat Cell Biol (2018) 20(7):775–81. 10.1038/s41556-018-0118-z PMC6030478 29941933

[120] Sullivan LB Luengo A Danai LV Bush LN Diehl FF Hosios AM. Aspartate is an Endogenous Metabolic Limitation for Tumour Growth. Nat Cell Biol (2018) 20(7):782–8. 10.1038/s41556-018-0125-0 PMC6051729 29941931

[121] Rabinovich S Adler L Yizhak K Sarver A Silberman A Agron S. Diversion of Aspartate in ASS1-Deficient Tumours Fosters De Novo Pyrimidine Synthesis. Nature (2015) 527(7578):379–83. 10.1038/nature15529.Citedin:Pubmed PMC4655447 26560030

[122] Sullivan LB Gui DY Hosios AM Bush LN Freinkman E Vander Heiden MG. Supporting Aspartate Biosynthesis Is an Essential Function of Respiration in Proliferating Cells. Cell (2015) 162(3):552–63. 10.1016/j.cell.2015.07.017 PMC4522278 26232225

[123] Borst P. The Malate-Aspartate Shuttle (Borst Cycle): How it Started and Developed Into a Major Metabolic Pathway. IUBMB Life (2020) 72(11):2241–59. 10.1002/iub.2367 PMC7693074 32916028

[124] Gaude E Schmidt C Gammage PA Dugourd A Blacker T Chew SP. NADH Shuttling Couples Cytosolic Reductive Carboxylation of Glutamine With Glycolysis in Cells With Mitochondrial Dysfunction. Mol Cell (2018) 69(4):581–593 e7. 10.1016/j.molcel.2018.01.034.Citedin:Pubmed 29452638 PMC5823973

[125] Thornburg JM Nelson KK Clem BF Lane AN Arumugam S Simmons A. Targeting Aspartate Aminotransferase in Breast Cancer. Breast Cancer Res (2008) 10(5):R84. 10.1186/bcr2154 18922152 PMC2614520

[126] Antti H Sellstedt M. Metabolic Effects of an Aspartate Aminotransferase-Inhibitor on Two T-Cell Lines. PloS One (2018) 13(12):e0208025. 10.1371/journal.pone.0208025 30532126 PMC6285999

[127] Yamada RH Wakabayashi Y Iwashima A Hasegawa T. Inhibition of Aspartate Aminotransferase by Hydrazinosuccinate. Biochim Biophys Acta (1984) 801(1):151–4. 10.1016/0304-4165(84)90224-1 6547859

[128] Yamada R Wakabayashi Y Iwashima A Hasegawa T. Inhibition of Aspartate Aminotransferase by D-Hydrazinosuccinate: Comparison With L-Hydrazinosuccinate. Biochim Biophys Acta (1986) 871(3):279–84. 10.1016/0167-4838(86)90209-8 3754770

[129] Yamada R Wakabayashi Y Iwashima A Hasegawa T. *In Vivo* Inhibition of Aspartate Aminotransferase in Mice by L-Hydrazinosuccinate. Biochim Biophys Acta (1987) 911(3):372–5. 10.1016/0167-4838(87)90080-x 3814611

[130] Holt MC Assar Z Beheshti Zavareh R Lin L Anglin J Mashadova O. Biochemical Characterization and Structure-Based Mutational Analysis Provide Insight Into the Binding and Mechanism of Action of Novel Aspartate Aminotransferase Inhibitors. Biochemistry (2018) 57(47):6604–14. 10.1021/acs.biochem.8b00914 PMC6487875 30365304

[131] Yoo HC Yu YC Sung Y Han JM. Glutamine Reliance in Cell Metabolism. Exp Mol Med (2020) 52(9):1496–516. 10.1038/s12276-020-00504-8 PMC8080614 32943735

[132] Xiang Y Stine ZE Xia J Lu Y O’Connor RS Altman BJ. Targeted Inhibition of Tumor-Specific Glutaminase Diminishes Cell-Autonomous Tumorigenesis. J Clin Invest. (2015) 125(6):2293–306. 10.1172/JCI75836 PMC4497742 25915584

[133] Gross MI Demo SD Dennison JB Chen L Chernov-Rogan T Goyal B. Antitumor Activity of the Glutaminase Inhibitor CB-839 in Triple-Negative Breast Cancer. Mol Cancer Ther (2014) 13(4):890–901. 10.1158/1535-7163.MCT-13-0870 24523301

[134] Boysen G Jamshidi-Parsian A Davis MA Siegel ER Simecka CM Kore RA. Glutaminase Inhibitor CB-839 Increases Radiation Sensitivity of Lung Tumor Cells and Human Lung Tumor Xenografts in Mice. Int J Radiat Biol (2019) 95(4):436–42. 10.1080/09553002.2018.1558299 PMC6622448 30557074

[135] Galan-Cobo A Sitthideatphaiboon P Qu X Poteete A Pisegna MA Tong P. LKB1 and KEAP1/NRF2 Pathways Cooperatively Promote Metabolic Reprogramming With Enhanced Glutamine Dependence in KRAS-Mutant Lung Adenocarcinoma. Cancer Res (2019) 79(13):3251–67. 10.1158/0008-5472.CAN-18-3527 PMC6606351 31040157

[136] Peterse EFP Niessen B Addie RD de Jong Y Cleven AHG Kruisselbrink AB. Targeting Glutaminolysis in Chondrosarcoma in Context of the IDH1/2 Mutation. Br J Cancer (2018) 118(8):1074–83. 10.1038/s41416-018-0050-9 PMC5931088 29576625

[137] Qie S Yoshida A Parnham S Oleinik N Beeson GC Beeson CC. Targeting Glutamine-Addiction and Overcoming CDK4/6 Inhibitor Resistance in Human Esophageal Squamous Cell Carcinoma. Nat Commun (2019) 10(1):1296. 10.1038/s41467-019-09179-w 30899002 PMC6428878

[138] Jin H Wang S Zaal EA Wang C Wu H Bosma A. A Powerful Drug Combination Strategy Targeting Glutamine Addiction for the Treatment of Human Liver Cancer. Elife (2020) 9. 10.7554/eLife.56749.Citedin:Pubmed PMC7535927 33016874

[139] Guerra V Dinardo CD Konopleva M Burger JA Borthakur G Jabbour E. Interim Results From a Phase Ib/II Clinical Study of the Glutaminase Inhibitor Telaglenastat (CB-839) in Combination With Azacitidine in Patients With Advanced Myelodysplastic Syndrome (MDS). J Clin Oncol (2019) 37(15_suppl):7037–7. 10.1200/JCO.2019.37.15_suppl.7037

[140] Wang JB Erickson JW Fuji R Ramachandran S Gao P Dinavahi R. Targeting Mitochondrial Glutaminase Activity Inhibits Oncogenic Transformation. Cancer Cell (2010) 18(3):207–19. 10.1016/j.ccr.2010.08.009 PMC3078749 20832749

[141] Yuan L Sheng X Clark LH Zhang L Guo H Jones HM. Glutaminase Inhibitor Compound 968 Inhibits Cell Proliferation and Sensitizes Paclitaxel in Ovarian Cancer. Am J Transl Res (2016) 8(10):4265–77. PMC5095319 27830010

[142] Katt WP Antonyak MA Cerione RA. Simultaneously Targeting Tissue Transglutaminase and Kidney Type Glutaminase Sensitizes Cancer Cells to Acid Toxicity and Offers New Opportunities for Therapeutic Intervention. Mol Pharm (2015) 12(1):46–55. 10.1021/mp500405h 25426679 PMC4291776

[143] Kalhan SC Hanson RW. Resurgence of Serine: An Often Neglected But Indispensable Amino Acid. J Biol Chem (2012) 287(24):19786–91. 10.1074/jbc.R112.357194 PMC3370164 22566694

[144] Serpa J. Cysteine as a Carbon Source, a Hot Spot in Cancer Cells Survival. Front Oncol (2020) 10:947. 10.3389/fonc.2020.00947 32714858 PMC7344258

[145] Maddocks ODK Athineos D Cheung EC Lee P Zhang T van den Broek NJF. Modulating the Therapeutic Response of Tumours to Dietary Serine and Glycine Starvation. Nature (2017) 544(7650):372–6. 10.1038/nature22056 28425994

[146] Maddocks OD Berkers CR Mason SM Zheng L Blyth K Gottlieb E. Serine Starvation Induces Stress and P53-Dependent Metabolic Remodelling in Cancer Cells. Nature (2013) 493(7433):542–6. 10.1038/nature11743 PMC6485472 23242140

[147] Tavana O Gu W. The Hunger Games: P53 Regulates Metabolism Upon Serine Starvation. Cell Metab (2013) 17(2):159–61. 10.1016/j.cmet.2013.01.012 PMC3634368 23395165

[148] Tajan M Hennequart M Cheung EC Zani F Hock AK Legrave N. Serine Synthesis Pathway Inhibition Cooperates With Dietary Serine and Glycine Limitation for Cancer Therapy. Nat Commun (2021) 12(1):366. 10.1038/s41467-020-20223-y 33446657 PMC7809039

[149] Jain M Nilsson R Sharma S Madhusudhan N Kitami T Souza AL. Metabolite Profiling Identifies a Key Role for Glycine in Rapid Cancer Cell Proliferation. Science (2012) 336(6084):1040–4. 10.1126/science.1218595 PMC3526189 22628656

[150] Combs JA DeNicola GM. The Non-Essential Amino Acid Cysteine Becomes Essential for Tumor Proliferation and Survival. Cancers (Basel) (2019) 11(5):678. 10.3390/cancers11050678.Citedin:Pubmed PMC6562400 31100816

[151] Nguyen CH Gluxam T Schlerka A Bauer K Grandits AM Hackl H. SOCS2 is Part of a Highly Prognostic 4-Gene Signature in AML and Promotes Disease Aggressiveness. Sci Rep (2019) 24 9(1):9139. 10.1038/s41598-019-45579-0 PMC6591510 31235852

[152] Bjelosevic S Gruber E Newbold A Shembrey C Devlin JR Hogg SJ. Serine Biosynthesis is a Metabolic Vulnerability in FLT3-ITD-Driven Acute Myeloid Leukaemia. Cancer Discov (2021) 11(6):1582–99. 10.1158/2159-8290.CD-20-0738.Citedin:Pubmed 33436370

[153] Yang B Wang C Xie Y Xu L Wu X Wu D. Monitoring Tyrosine Kinase Inhibitor Therapeutic Responses With a Panel of Metabolic Biomarkers in Chronic Myeloid Leukemia Patients. Cancer Sci (2018) 109(3):777–84. 10.1111/cas.13500 PMC5834806 29316075

[154] Karlikova R Siroka J Friedecky D Faber E Hrda M Micova K. Metabolite Profiling of the Plasma and Leukocytes of Chronic Myeloid Leukemia Patients. J Proteome Res (2016) 15(9):3158–66. 10.1021/acs.jproteome.6b00356 27465658

[155] Hattori A Tsunoda M Konuma T Kobayashi M Nagy T Glushka J. Cancer Progression by Reprogrammed BCAA Metabolism in Myeloid Leukaemia. Nature (2017) 545(7655):500–4. 10.1038/nature22314 PMC5554449 28514443

[156] Polet F Corbet C Pinto A Rubio LI Martherus R Bol V. Reducing the Serine Availability Complements the Inhibition of the Glutamine Metabolism to Block Leukemia Cell Growth. Oncotarget (2016) 7(2):1765–76. 10.18632/oncotarget.6426 PMC4811496 26625201

[157] Regan JD Vodopick N Takeda S Lee WH Faulcon FM. Serine Requirement in Leukemic and Normal Blood Cells. Science (1969) 163(3874):1452–3. 10.1126/science.163.3874.1452 5251122

[158] Onuma T Waligunda J Holland JF. Amino Acid Requirements *In Vitro* of Human Leukemic Cells. Cancer Res (1971) 31(11):1640–4. 5287343

[159] Wang D Yang H Zhang Y Hu R Hu D Wang Q. Inhibition of Cystathionine Beta-Synthase Promotes Apoptosis and Reduces Cell Proliferation in Chronic Myeloid Leukemia. Signal Transduct Target Ther (2021) 6(1):52. 10.1038/s41392-020-00410-5 33558454 PMC7870845

[160] Kardos GR Wastyk HC Robertson GP. Disruption of Proline Synthesis in Melanoma Inhibits Protein Production Mediated by the GCN2 Pathway. Mol Cancer Res (2015) 13(10):1408. 10.1158/1541-7786.MCR-15-0048 26082174 PMC5238710

[161] Loayza-Puch F Rooijers K Buil LCM Zijlstra J F. Oude Vrielink J Lopes R. Tumour-Specific Proline Vulnerability Uncovered by Differential Ribosome Codon Reading. Nature (2016) 530(7591):490–4. 10.1038/nature16982 26878238

[162] Ding J Kuo M-L Su L Xue L Luh F Zhang H. Human Mitochondrial Pyrroline-5-Carboxylate Reductase 1 Promotes Invasiveness and Impacts Survival in Breast Cancers. Carcinogenesis (2017) 38(5):519–31. 10.1093/carcin/bgx022 28379297

[163] Christensen EM Bogner AN Vandekeere A Tam GS Patel SM Becker DF. In Crystallo Screening for Proline Analog Inhibitors of the Proline Cycle Enzyme PYCR1. J Biol Chem (2020) 295(52):18316–27. 10.1074/jbc.RA120.016106 PMC7939384 33109600

[164] Milne K Sun J Zaal EA Mowat J Celie PHN Fish A. A Fragment-Like Approach to PYCR1 Inhibition. Bioorg Med Chem Lett (2019) 29(18):2626–31. 10.1016/j.bmcl.2019.07.047 31362921

[165] Jones CL Stevens BM D’Alessandro A Reisz JA Culp-Hill R Nemkov T. Inhibition of Amino Acid Metabolism Selectively Targets Human Leukemia Stem Cells. Cancer Cell (2019) 35(2):333–5. 10.1016/j.ccell.2019.01.013 PMC6389327 30753831

[166] Stockard B Garrett T Guingab-Cagmat J Meshinchi S Lamba J. Distinct Metabolic Features Differentiating FLT3-ITD AML From FLT3-WT Childhood Acute Myeloid Leukemia. Sci Rep (2018) 8(1):5534. 10.1038/s41598-018-23863-9 29615816 PMC5882915

[167] Fenouille N Bassil CF Ben-Sahra I Benajiba L Alexe G Ramos A. The Creatine Kinase Pathway is a Metabolic Vulnerability in EVI1-Positive Acute Myeloid Leukemia. Nat Med (2017) 23(3):301–13. 10.1038/nm.4283 PMC5540325 28191887

[168] Felig P Pozefsky T Marliss E Cahill GF Jr. Alanine: Key Role in Gluconeogenesis. Science (1970) 167(3920):1003–4. 10.1126/science.167.3920.1003 5411169

[169] Hewton KG Johal AS Parker SJ. Transporters at the Interface Between Cytosolic and Mitochondrial Amino Acid Metabolism. Metabolites (2021) 11(2):112. 10.3390/metabo11020112.Citedin:Pubmed 33669382 PMC7920303

[170] Sousa CM Biancur DE Wang X Halbrook CJ Sherman MH Zhang L. Pancreatic Stellate Cells Support Tumour Metabolism Through Autophagic Alanine Secretion. Nature (2016) 536(7617):479–83. 10.1038/nature19084 PMC5228623 27509858

[171] Xu R Yang J Ren B Wang H Yang G Chen Y. Reprogramming of Amino Acid Metabolism in Pancreatic Cancer: Recent Advances and Therapeutic Strategies. Front Oncol (2020) 10:572722. 10.3389/fonc.2020.572722 33117704 PMC7550743

[172] Elia I Rossi M Stegen S Broekaert D Doglioni G van Gorsel M. Breast Cancer Cells Rely on Environmental Pyruvate to Shape the Metastatic Niche. Nature (2019) 568(7750):117–21. 10.1038/s41586-019-0977-x PMC6451642 30814728

[173] Weinberg F Hamanaka R Wheaton WW Weinberg S Joseph J Lopez M. Mitochondrial Metabolism and ROS Generation are Essential for Kras-Mediated Tumorigenicity. Proc Natl Acad Sci U S A (2010) 107(19):8788–93. 10.1073/pnas.1003428107 PMC2889315 20421486

[174] Ye J Kumanova M Hart LS Sloane K Zhang H De Panis DN. The GCN2-ATF4 Pathway is Critical for Tumour Cell Survival and Proliferation in Response to Nutrient Deprivation. EMBO J (2010) 29(12):2082–96. 10.1038/emboj.2010.81 PMC2892366 20473272

[175] Liu K Sutter BM Tu BP. Autophagy Sustains Glutamate and Aspartate Synthesis in Saccharomyces Cerevisiae During Nitrogen Starvation. Nat Commun (2021) 12(1):57. 10.1038/s41467-020-20253-6 33397945 PMC7782722

[176] Hinnebusch AG. Translational Regulation of GCN4 and the General Amino Acid Control of Yeast. Annu Rev Microbiol (2005) 59:407–50. 10.1146/annurev.micro.59.031805.133833 16153175

[177] Chantranupong L Wolfson RL Sabatini DM. Nutrient-Sensing Mechanisms Across Evolution. Cell (2015) 161(1):67–83. 10.1016/j.cell.2015.02.041 25815986 PMC4384161

[178] Carroll B Korolchuk VI Sarkar S. Amino Acids and Autophagy: Cross-Talk and Co-Operation to Control Cellular Homeostasis. Amino Acids (2015) 47(10):2065–88. 10.1007/s00726-014-1775-2 24965527

[179] Kilberg MS Shan J Su N. ATF4-Dependent Transcription Mediates Signaling of Amino Acid Limitation. Trends Endocrinol Metab (2009) 20(9):436–43. 10.1016/j.tem.2009.05.008 PMC3587693 19800252

[180] Krishnamoorthy T Pavitt GD Zhang F Dever TE Hinnebusch AG. Tight Binding of the Phosphorylated Alpha Subunit of Initiation Factor 2 (Eif2alpha) to the Regulatory Subunits of Guanine Nucleotide Exchange Factor Eif2b is Required for Inhibition of Translation Initiation. Mol Cell Biol (2001) 21(15):5018–30. 10.1128/MCB.21.15.5018-5030.2001 PMC87228 11438658

[181] Hinnebusch AG Ivanov IP Sonenberg N. Translational Control by 5’-Untranslated Regions of Eukaryotic mRNAs. Science (2016) 352(6292):1413–6. 10.1126/science.aad9868 PMC7422601 27313038

[182] Chikashige Y Kato H Thornton M Pepper W Hilgers M Cecil A. Gcn2 Eif2alpha Kinase Mediates Combinatorial Translational Regulation Through Nucleotide Motifs and uORFs in Target mRNAs. Nucleic Acids Res (2020) 48(16):8977–92. 10.1093/nar/gkaa608 PMC7498311 32710633

[183] Leppek K Das R Barna M. Functional 5’ UTR mRNA Structures in Eukaryotic Translation Regulation and How to Find Them. Nat Rev Mol Cell Biol (2018) 19(3):158–74. 10.1038/nrm.2017.103 PMC5820134 29165424

[184] Lindqvist LM Tandoc K Topisirovic I Furic L. Cross-Talk Between Protein Synthesis, Energy Metabolism and Autophagy in Cancer. Curr Opin Genet Dev (2018) 48:104–11. 10.1016/j.gde.2017.11.003 PMC5869074 29179096

[185] B’Chir W Maurin AC Carraro V Averous J Jousse C Muranishi Y. The Eif2alpha/ATF4 Pathway is Essential for Stress-Induced Autophagy Gene Expression. Nucleic Acids Res (2013) 41(16):7683–99. 10.1093/nar/gkt563 PMC3763548 23804767

[186] Harding HP Zhang Y Zeng H Novoa I Lu PD Calfon M. An Integrated Stress Response Regulates Amino Acid Metabolism and Resistance to Oxidative Stress. Mol Cell (2003) 11(3):619–33. 10.1016/s1097-2765(03)00105-9 12667446

[187] Saxton RA Sabatini DM. mTOR Signaling in Growth, Metabolism, and Disease. Cell (2017) 168(6):960–76. 10.1016/j.cell.2017.02.004 PMC5394987 28283069

[188] Shimobayashi M Hall MN. Multiple Amino Acid Sensing Inputs to Mtorc1. Cell Res (2016) 26(1):7–20. 10.1038/cr.2015.146 26658722 PMC4816134

[189] Kitada M Xu J Ogura Y Monno I Koya D. Mechanism of Activation of Mechanistic Target of Rapamycin Complex 1 by Methionine. Front Cell Dev Biol (2020) 8:715. 10.3389/fcell.2020.00715 32850834 PMC7431653

[190] Wolfson RL Sabatini DM. The Dawn of the Age of Amino Acid Sensors for the Mtorc1 Pathway. Cell Metab (2017) 26(2):301–9. 10.1016/j.cmet.2017.07.001 PMC5560103 28768171

[191] Takahara T Amemiya Y Sugiyama R Maki M Shibata H. Amino Acid-Dependent Control of Mtorc1 Signaling: A Variety of Regulatory Modes. J BioMed Sci (2020) 27(1):87. 10.1186/s12929-020-00679-2 32799865 PMC7429791

[192] Hua H Kong Q Zhang H Wang J Luo T Jiang Y. Targeting mTOR for Cancer Therapy. J Hematol Oncol (2019) 12(1):71. 10.1186/s13045-019-0754-1 31277692 PMC6612215

[193] Musa J Orth MF Dallmayer M Baldauf M Pardo C Rotblat B. Eukaryotic Initiation Factor 4E-Binding Protein 1 (4E-BP1): A Master Regulator of mRNA Translation Involved in Tumorigenesis. Oncogene (2016) 35(36):4675–88. 10.1038/onc.2015.515 26829052

[194] Martelli AM Evangelisti C Chappell W Abrams SL Basecke J Stivala F. Targeting the Translational Apparatus to Improve Leukemia Therapy: Roles of the PI3K/PTEN/Akt/mTOR Pathway. Leukemia (2011) 25(7):1064–79. 10.1038/leu.2011.46 21436840

[195] Moschetta M Reale A Marasco C Vacca A Carratu MR. Therapeutic Targeting of the mTOR-Signalling Pathway in Cancer: Benefits and Limitations. Br J Pharmacol (2014) 171(16):3801–13. 10.1111/bph.12749 PMC4128044 24780124

[196] Mugume Y Kazibwe Z Bassham DC. Target of Rapamycin in Control of Autophagy: Puppet Master and Signal Integrator. Int J Mol Sci (2020) 21(21):8259. 10.3390/ijms21218259.Citedin:Pubmed PMC7672647 33158137

[197] Dossou AS Basu A. The Emerging Roles of Mtorc1 in Macromanaging Autophagy. Cancers (Basel) (2019) 11(10):1422. 10.3390/cancers11101422.Citedin:Pubmed PMC6826502 31554253

[198] Rabanal-Ruiz Y Korolchuk VI. Mtorc1 and Nutrient Homeostasis: The Central Role of the Lysosome. Int J Mol Sci (2018) 19(3):818. 10.3390/ijms19030818.Citedin:Pubmed PMC5877679 29534520

[199] Rabanal-Ruiz Y Otten EG Korolchuk VI. Mtorc1 as the Main Gateway to Autophagy. Essays Biochem (2017) 61(6):565–84. 10.1042/EBC20170027 PMC5869864 29233869

[200] Noda T. Regulation of Autophagy Through TORC1 and Mtorc1. Biomolecules (2017) 7(3):52. 10.3390/biom7030052 PMC5618233 28686223

[201] Dennis MD McGhee NK Jefferson LS Kimball SR. Regulated in DNA Damage and Development 1 (REDD1) Promotes Cell Survival During Serum Deprivation by Sustaining Repression of Signaling Through the Mechanistic Target of Rapamycin in Complex 1 (Mtorc1). Cell Signal (2013) 25(12):2709–16. 10.1016/j.cellsig.2013.08.038 PMC3867791 24018049

[202] Minami K Tambe Y Watanabe R Isono T Haneda M Isobe K. Suppression of Viral Replication by Stress-Inducible GADD34 Protein *via* the Mammalian Serine/Threonine Protein Kinase mTOR Pathway. J Virol (2007) 81(20):11106–15. 10.1128/JVI.01063-07 PMC2045534 17670836

[203] Averous J Lambert-Langlais S Mesclon F Carraro V Parry L Jousse C. GCN2 Contributes to Mtorc1 Inhibition by Leucine Deprivation Through an ATF4 Independent Mechanism. Sci Rep (2016) 6:27698. 10.1038/srep27698 27297692 PMC4906353

[204] Fukuda T Sofyantoro F Tai YT Chia KH Matsuda T Murase T. Tripartite Suppression of Fission Yeast TORC1 Signaling by the GATOR1-Sea3 Complex, the TSC Complex, and Gcn2 Kinase. Elife (2021) 10. 10.7554/eLife.60969.Citedin:Pubmed PMC7857730 33534698

[205] Ye J Palm W Peng M King B Lindsten T Li MO. GCN2 Sustains Mtorc1 Suppression Upon Amino Acid Deprivation by Inducing Sestrin2. Genes Dev (2015) 29(22):2331–6. 10.1101/gad.269324.115 PMC4691887 26543160

[206] Nikonorova IA Mirek ET Signore CC Goudie MP Wek RC Anthony TG. Time-Resolved Analysis of Amino Acid Stress Identifies Eif2 Phosphorylation as Necessary to Inhibit Mtorc1 Activity in Liver. J Biol Chem (2018) 293(14):5005–15. 10.1074/jbc.RA117.001625 PMC5892569 29449374

[207] Wengrod J Wang D Weiss S Zhong H Osman I Gardner LB. Phosphorylation of Eif2alpha Triggered by Mtorc1 Inhibition and PP6C Activation is Required for Autophagy and is Aberrant in PP6C-Mutated Melanoma. Sci Signal (2015) 8(367):ra27. 10.1126/scisignal.aaa0899.Citedin:Pubmed 25759478 PMC4580977

[208] Nakamura A Nambu T Ebara S Hasegawa Y Toyoshima K Tsuchiya Y. Inhibition of GCN2 Sensitizes ASNS-Low Cancer Cells to Asparaginase by Disrupting the Amino Acid Response. Proc Natl Acad Sci U S A (2018) 115(33):E7776–85. 10.1073/pnas.1805523115 PMC6099884 30061420

[209] Broer A Gauthier-Coles G Rahimi F van Geldermalsen M Dorsch D Wegener A. Ablation of the ASCT2 (SLC1A5) Gene Encoding a Neutral Amino Acid Transporter Reveals Transporter Plasticity and Redundancy in Cancer Cells. J Biol Chem (2019) 294(11):4012–26. 10.1074/jbc.RA118.006378 PMC6422075 30635397

[210] Bohlen J Harbrecht L Blanco S Clemm von Hohenberg K Fenzl K Kramer G. DENR Promotes Translation Reinitiation *via* Ribosome Recycling to Drive Expression of Oncogenes Including ATF4. Nat Commun (2020) 11(1):4676. 10.1038/s41467-020-18452-2 32938922 PMC7494916

[211] Parzych K Saavedra-Garcia P Valbuena GN Al-Sadah HA Robinson ME Penfold L. The Coordinated Action of VCP/p97 and GCN2 Regulates Cancer Cell Metabolism and Proteostasis During Nutrient Limitation. Oncogene (2019) 38(17):3216–31. 10.1038/s41388-018-0651-z PMC6756015 30626938

[212] Kato Y Kunimasa K Takahashi M Harada A Nagasawa I Osawa M. GZD824 Inhibits GCN2 and Sensitizes Cancer Cells to Amino Acid Starvation Stress. Mol Pharmacol (2020) 98(6):669–76. 10.1124/molpharm.120.000070 33033108

[213] Wang SF Chen MS Chou YC Ueng YF Yin PH Yeh TS. Mitochondrial Dysfunction Enhances Cisplatin Resistance in Human Gastric Cancer Cells *via* the ROS-Activated GCN2-Eif2alpha-ATF4-xCT Pathway. Oncotarget (2016) 7(45):74132–51. 10.18632/oncotarget.12356 PMC5342041 27708226

[214] Longchamp A Mirabella T Arduini A MacArthur MR Das A Trevino-Villarreal JH. Amino Acid Restriction Triggers Angiogenesis *via* GCN2/ATF4 Regulation of VEGF and H2S Production. Cell (2018) 173(1):117–129 e14. 10.1016/j.cell.2018.03.001.Citedin:Pubmed 29570992 PMC5901681

[215] Koppula P Zhang Y Zhuang L Gan B. Amino Acid Transporter SLC7A11/xCT at the Crossroads of Regulating Redox Homeostasis and Nutrient Dependency of Cancer. Cancer Commun (Lond) (2018) 38(1):12. 10.1186/s40880-018-0288-x 29764521 PMC5993148

[216] Gremke N Polo P Dort A Schneikert J Elmshauser S Brehm C. mTOR-Mediated Cancer Drug Resistance Suppresses Autophagy and Generates a Druggable Metabolic Vulnerability. Nat Commun (2020) 11(1):4684. 10.1038/s41467-020-18504-7 32943635 PMC7499183

[217] Wang Z Xie Q Zhou H Zhang M Shen J Ju D. Amino Acid Degrading Enzymes and Autophagy in Cancer Therapy. Front Pharmacol (2020) 11:582587. 10.3389/fphar.2020.582587 33510635 PMC7836011

[218] Takahashi H Inoue J Sakaguchi K Takagi M Mizutani S Inazawa J. Autophagy is Required for Cell Survival Under L-Asparaginase-Induced Metabolic Stress in Acute Lymphoblastic Leukemia Cells. Oncogene (2017) 36(30):4267–76. 10.1038/onc.2017.59 PMC5537607 28346428

[219] Polak R Bierings MB van der Leije CS Sanders MA Roovers O Marchante JRM. Autophagy Inhibition as a Potential Future Targeted Therapy for ETV6-RUNX1-Driven B-Cell Precursor Acute Lymphoblastic Leukemia. Haematologica (2019) 104(4):738–48. 10.3324/haematol.2018.193631 PMC6442983 30381299

[220] Chen Q Ye L Fan J Zhang X Wang H Liao S. Autophagy Suppression Potentiates the Anti-Glioblastoma Effect of Asparaginase *In Vitro* and *In Vivo* . Oncotarget (2017) 8(53):91052–66. 10.18632/oncotarget.19409.Citedin:Pubmed PMC5710905 29207624

[221] Ji Y Li L Tao Q Zhang X Luan J Zhao S. Deprivation of Asparagine Triggers Cytoprotective Autophagy in Laryngeal Squamous Cell Carcinoma. Appl Microbiol Biotechnol (2017) 101(12):4951–61. 10.1007/s00253-017-8221-9 28352997

[222] Zhang B Fan J Zhang X Shen W Cao Z Yang P. Targeting Asparagine and Autophagy for Pulmonary Adenocarcinoma Therapy. Appl Microbiol Biotechnol (2016) 100(21):9145–61. 10.1007/s00253-016-7640-3 27251546

[223] Delage B Luong P Maharaj L O’Riain C Syed N Crook T. Promoter Methylation of Argininosuccinate Synthetase-1 Sensitises Lymphomas to Arginine Deiminase Treatment, Autophagy and Caspase-Dependent Apoptosis. Cell Death Dis (2012) 3:e342. 10.1038/cddis.2012.83 22764101 PMC3406582

[224] Savaraj N You M Wu C Wangpaichitr M Kuo MT Feun LG. Arginine Deprivation, Autophagy, Apoptosis (AAA) for the Treatment of Melanoma. Curr Mol Med (2010) 10(4):405–12. 10.2174/156652410791316995 PMC3096550 20459375

[225] Kim RH Coates JM Bowles TL McNerney GP Sutcliffe J Jung JU. Arginine Deiminase as a Novel Therapy for Prostate Cancer Induces Autophagy and Caspase-Independent Apoptosis. Cancer Res (2009) 69(2):700–8. 10.1158/0008-5472.CAN-08-3157 PMC2629384 19147587

[226] Wang Z Shi X Li Y Fan J Zeng X Xian Z. Blocking Autophagy Enhanced Cytotoxicity Induced by Recombinant Human Arginase in Triple-Negative Breast Cancer Cells. Cell Death Dis (2014) 5:e1563. 10.1038/cddis.2014.503 25501824 PMC4454157

[227] Li Y Zeng X Wang S Fan J Wang Z Song P. Blocking Autophagy Enhanced Leukemia Cell Death Induced by Recombinant Human Arginase. Tumour Biol (2016) 37(5):6627–35. 10.1007/s13277-015-4253-x 26643895

[228] Lin C Wang Z Li L He Y Fan J Liu Z. The Role of Autophagy in the Cytotoxicity Induced by Recombinant Human Arginase in Laryngeal Squamous Cell Carcinoma. Appl Microbiol Biotechnol (2015) 99(20):8487–94. 10.1007/s00253-015-6565-6 25904129

[229] Shen W Zhang X Fu X Fan J Luan J Cao Z. A Novel and Promising Therapeutic Approach for NSCLC: Recombinant Human Arginase Alone or Combined With Autophagy Inhibitor. Cell Death Dis (2017) 8(3):e2720. 10.1038/cddis.2017.137 28358368 PMC5386540

[230] Li J Song P Zhu L Aziz N Zhou Q Zhang Y. Synthetic Lethality of Glutaminolysis Inhibition, Autophagy Inactivation and Asparagine Depletion in Colon Cancer. Oncotarget (2017) 8(26):42664–72. 10.18632/oncotarget.16844 PMC5522096 28424408

[231] Kandasamy P Gyimesi G Kanai Y Hediger MA. Amino Acid Transporters Revisited: New Views in Health and Disease. Trends Biochem Sci (2018) 43(10):752–89. 10.1016/j.tibs.2018.05.003 30177408

[232] Wei Z Liu X Cheng C Yu W Yi P. Metabolism of Amino Acids in Cancer. Front Cell Dev Biol (2020) 8:603837. 10.3389/fcell.2020.603837 33511116 PMC7835483

[233] Lopes C Pereira C Medeiros R. ASCT2 and LAT1 Contribution to the Hallmarks of Cancer: From a Molecular Perspective to Clinical Translation. Cancers (Basel) (2021) 13(2):203. 10.3390/cancers13020203.Citedin:Pubmed PMC7828050 33429909

[234] Ganapathy V Thangaraju M Prasad PD. Nutrient Transporters in Cancer: Relevance to Warburg Hypothesis and Beyond. Pharmacol Ther (2009) 121(1):29–40. 10.1016/j.pharmthera.2008.09.005 18992769

[235] Lin W Wang C Liu G Bi C Wang X Zhou Q. SLC7A11/xCT in Cancer: Biological Functions and Therapeutic Implications. Am J Cancer Res (2020) 10(10):3106–26. PMC7642655 33163260

[236] Bacci M Lorito N Ippolito L Ramazzotti M Luti S Romagnoli S. Reprogramming of Amino Acid Transporters to Support Aspartate and Glutamate Dependency Sustains Endocrine Resistance in Breast Cancer. Cell Rep (2019) 28(1):104–18.e8. 10.1016/j.celrep.2019.06.010.Citedin:Pubmed 31269432 PMC6616584

